# Investigating genetic modifications to enhance L1CAM-CAR T cell migration in solid tumors in a 3D bioprinted neuroblastoma model

**DOI:** 10.3389/fimmu.2025.1677361

**Published:** 2025-11-27

**Authors:** Lena Andersch, Laura Grunewald, Maria Stecklum, Filippos Klironomos, Kerstin Haase, Viola Hollek, Tobias Lam, Beate Anahita Jung, Anika Winkler, Silke Schwiebert, Kathy Astrahantseff, Michael Launspach, Marvin Jens, Anton Henssen, Lutz Kloke, Nils Blüthgen, Angelika Eggert, Johannes H. Schulte, Kathleen Anders, Annette Künkele

**Affiliations:** 1 German Cancer Research Center (DKFZ), Heidelberg, Germany; 2 Department of Pediatric Oncology and Hematology, Charité – Universitätsmedizin Berlin, Corporate Member of Freie Universität Berlin, Humboldt-Universität zu Berlin, and Berlin Institute of Health, Berlin, Germany; 3 German Cancer Consortium (DKTK), Partner Site Berlin, Charité Comprehensive Cancer Center (CCCC), Berlin, Germany; 4 Experimental Pharmacology and Oncology (EPO) Berlin-Buch GmbH, Berlin, Germany; 5 Institute of Pathology, Charité - Universitätsmedizin Berlin, Corporate Member of Freie Universität Berlin and Humboldt-Universität zu Berlin, Berlin, Germany; 6 Institute for Biology, Humboldt-Universität zu Berlin, Berlin, Germany; 7 Cellbricks GmbH Berlin, Berlin, Germany

**Keywords:** migration, CAR T cells, neuroblastoma, SELPLG, SH2D2A

## Abstract

**Introduction:**

Effective CAR T cell infiltration into solid tumors remains a major barrier to therapy success. Despite their clinical potential, few studies have evaluated phenotypes of CAR T cells successfully invading the tumor mass following infusion. Phenotypic information would enrich our understanding of the mechanisms governing CAR T cell migration into solid tumors. Here we implemented an *in vitro* strategy to identify genes driving L1CAM-CAR T cell migration into a 3D tumor mass.

**Methods:**

L1CAM-CAR T cells were separated into 2 groups by their capability to infiltrate (or not) a 3D bioprinted neuroblastoma model. Single-cell and bulk RNA sequencing was performed, and infiltrating CAR T cells were compared to noninfiltrating cells to seek genetic drivers of CAR T cell migration. CRISPR/Cas9 technology was used to generate modified L1CAM-CAR T cells.

**Results:**

Tumor-infiltrating L1CAM-CAR T cells expressed lower levels of the selectin P ligand (SELPLG) glycoprotein and higher levels of the T cell-specific adaptor protein, SH2D2A. Functional characterization of L1CAM-CAR T cells genetically modified to enforce these characteristics demonstrated that neither trait negatively impacted L1CAM-CAR T cell cytotoxicity, activation and cytokine release upon coculture with neuroblastoma target cells. Transgenic SH2D2A expression did not improve CAR T cell migration in an endothelial transmembrane assay. SELPLG knockout benefited CAR T cell *in vitro* trans-endothelial migration, but did not enhance anti-tumor efficacy in an immunodeficient mouse model.

**Discussion:**

Our findings reveal a key limitation of murine xenograft models, which are widely used as the gold standard for preclinical CAR T cell testing. The lack of conservation between the human and murine SELPLG proteins likely accounts for the discrepancy between enhanced *in vitro* migration of SELPLG-deficient L1CAM-CAR T cells and their lack of improved efficacy in the mouse model. This underscores the need for more predictive human-relevant models to better preclinically evaluate CAR T cell function.

## 1 Introduction

Neuroblastoma is the most common extracranial tumor in children (1). Despite significant advancements in treatment, the prognosis for patients with high-risk disease remains poor, with < 50% surviving 5 years (2). Immunotherapy in the form of monoclonal antibodies targeting the GD2 disialoganglioside on the neuroblastoma cell surface has entered trials for first-line treatment regimens (NCT03363373, NCT02914405). Building on this approach, chimeric antigen receptor (CAR) T cells were developed to target GD2, and were the first CAR T cells tested in early clinical trials for children with neuroblastoma (3). Disappointingly, their clinical success has been limited particularly against cases with high tumor load (4).

The first challenge CAR T cells face is to infiltrate the solid tumor mass (4–6). To extravasate from the blood stream and enter the tumor site, (CAR) T cells sense chemokine gradients including CXCL9, CXCL10 or CXCL11, which are released by tumor-related stromal and endothelial cells and presented on the endothelial surface. These chemokines bind to specific receptors, such as CXCR3 on T cells, which also interact with selectins (P and E) on the endothelium, slowing (CAR) T cell movement and initiating rolling. Further binding to adhesion molecules such as ICAM and VCAM, via integrin subunits, supports firm adhesion and induces cell arrest (7–9). Transmigration through the endothelium depends on the ability to constantly remodel T cell shape, which is coordinated by integrating motility signals with actin cytoskeletal reorganization [e.g. actin-myosin contractions (10, 11)]. Once within the extracellular matrix, migration is directed by the balance in signals mediating “stop” (adhesion- or TCR signaling-related) and “go” [chemokine-mediated (12)].

Increased matrix cross-linking, fiber alignment and collagen deposition in solid tumors stiffens the extracellular matrix (ECM), and further impedes (CAR) T cell movement through the tumor ECM. Our understanding of CAR T cells that successfully migrate through the surrounding tissue stroma and enter the solid tumor remains limited, since only a few studies have characterized CAR T cells isolated from tumors after infusion (13–15). While circulating CAR T cells isolated from the blood are regularly analyzed (4, 15, 16), particularly in pediatric patients, performing consecutive tumor biopsies is challenging and not ethical in cases that primarily provide diagnostic material or treatment for the patient. This data gap underlies several unknowns in the field. We do not know what characteristics CAR T cells that reach the tumor site specifically have or how many CAR T cells typically enter a tumor. Whether CAR T cells proliferate or persist once they enter the tumor or how long they remain functional are also unclear. This data gap in the field could be filled by evaluating the phenotype and activity of tumor-infiltrating CAR T cells.

Here we implemented a structured *in vitro* strategy, seeking genetic drivers of CAR T cell migration into and through a 3D tumor mass. We utilized neuroblastoma-specific L1CAM-CAR T cells, which we have developed and tested in an early clinical trial [(17) NCT02311621], and an established 3D bioprinted neuroblastoma model (18) for coculture experiments. The model consists of SK-N-BE (2) neuroblastoma cells resuspended in methacrylated gelatin and stereolithographically printed as reproducible 4-mm (diameter) tumor masses. L1CAM- CAR T cells capable of infiltrating the tumor model were separately analyzed using single-cell and bulk RNA sequencing, and compared to CAR T cells that did not enter the 3D tumor model. We genetically modified differentially regulated genes in L1CAM-CAR T cells to validate their impact on CAR T cell function and migratory capacity *in vitro* and *in vivo*.

## 2 Materials and methods

### 2.1 *In vivo* studies in mice

Housing, handling and all mouse experiments were approved by the regulatory agency (Landesamt für Gesundheit und Soziales Berlin, approval number: Reg 0010/19) and were carried out in compliance with the German Law of Animal Rights. Food and water were provided ad libitum to mice housed at 23°C in a 12 h light/dark cycle. Animal welfare was checked twice daily. Body weights, tumor volume and general health conditions were recorded throughout the study. EPO Berlin-Buch GmbH conducted animal experiments in 6- to 8-week old female CIEA NOG^®^ mice (nomenclature: NOD.Cg-Prkdcscid Il2rgtm1Sug/JicTac; genotype: sp/sp;ko/ko; Taconic Biosciences, Inc.). NOG mice were subcutaneously injected with 5x10^6^ SK-N-AS tumor cells resuspended in a 50% matrigel in phosphate-buffered saline. L1CAM-CAR T cells (1 x 10^6^) expressing the firefly luciferase gene were injected on day 7. CAR T cell expansion and tumor homing was analyzed in anesthetized mice via bioluminescent imaging with the NightOwl II LB983 *in vivo* imaging system at the indicated times. Mice were anesthetized with isoflurane (Baxter, San Juan, Puerto Rico) and intraperitoneally injected with 150 mg/kg D-luciferin (Biosynth, Staad, Switzerland) dissolved in PBS for imaging. IndiGO 2.0.5.0 software was used for initial analysis, color-coding the signal intensity and quantification. Tumor size was measured by an electronic caliper along 3 orthogonal axes (a, b and c), and tumor volumes were calculated by V(mm^3^) = (a × b × c)/2.

### 2.2 CAR construct and CAR T cell generation

The previously described L1CAM-specific CE7-CAR (19) was cloned into the SIN epHIV7 lentiviral vector then propagated in 293T cells and isolated as previously described (20). The single-chain variable fragment (scFv) in the CAR construct was codon optimized and subsequently linked to a 12-amino acid spacer domain from the human IgG4-Fc hinge. The spacer domain connects the antigen-binding domain to the CD28 transmembrane domain followed by the CD3zeta (ζ) signaling module and either the CD28 or 4-1BB cytoplasmic costimulatory domain (second generation CAR). CAR constructs were linked downstream to a T2A self-cleaving peptide and a truncated epidermal growth factor receptor (EGFRt), allowing CAR T cell detection and enrichment (21). T cells were lentivirally transduced on day one after activation with a multiplicity of infection (MOI) of 1. SELPLG knockout was generated in the L1CAM-CAR T cells (harboring CD28 costimulatory domain, day 2 after lentiviral transfection) by nucleofecting SELPLG-specific guide RNA using the P3 Primary T cell nucleofection kit (cat# V4XP-3032, Lonza) according to manufacturer instructions. Briefly, the EO115 program of the 4D nucleofector (Lonza) was used to nucleofect 1 x 10^6^ T cells resuspended in 20 µl P3 buffer and 1.38 μl (100 μM) precomplexed CRISPR-Cas9 ribonucleoproteins consisting of synthetically modified single guide RNA (sgRNA, Integrated DNA Technologies, 15–50 kDa 100 μg/μl), 15–50 kDa poly(l-glutamic acid) (Sigma) and recombinant SpCas9 protein (61 μM, cat#1081058 Integrated DNATechnologies) in a 0.96:1:0.8 volume ratio. Cells were expanded in T cell medium (changed every 2–3 days) supplemented with 0.5 ng/ml IL15 (cat# 130-095-765, Miltenyi Biotec) and 5 ng/ml IL7 (cat# 130-095-362, Miltenyi Biotec). Knockout efficiency was analyzed by immunostaining with anti-SELPLG antibody before flow cytometric analysis. CAR T cells were generated from healthy donors (Charité ethics committee approval EA2/262/20) as previously described (19). T cells used as controls alongside CAR T cells in experiments were not lentivirally transduced. CAR and control T cells were cryopreserved until further use. Cryopreserved cells were thawed and stimulated with irradiated peripheral blood mononuclear cells, irradiated CD19^+^ EBV-transformed lymphoblastoid cell line (TM-LCL) and 30ng/mL antibody activating the CD3 complex (OKT3 clone, cat#130-093-387, Miltenyi Biotec). For rapid expansion, T cells were maintained in RPMI 1640 media supplemented with 10% fetal calf serum, 0.5ng/mL IL15 (cat# 130-095-765, Miltenyi Biotec) and 5ng/ml IL7 (cat# 130-095-362, Miltenyi Biotec) according to a rapid expansion protocol (22). Functional *in vitro* assays were conducted between days 11 and 16 of culture.

### 2.3 Neuroblastoma cell culture

SK-N-BE (2) neuroblastoma cells (passaged ≤20 times from stock cultures expanded in <10 passages from the source culture obtained from ATCC) were propagated in Dulbecco´s Modified Eagle Medium (Life Technologies, Karlsbad, CA, USA) supplemented with 10% heat-inactivated fetal calf serum (Sigma-Aldrich, St. Louis, MO, USA) to 80% density in 2D culture before 3D bioprinting or seeding for functional assays. The identity of the SK-N-BE (2) neuroblastoma cell line was confirmed by Eurofins (Luxemburg) and *Mycoplasma*-negative by a cell-based colometric HEK-Blue Detection assay (Invivogen).

### 2.4 3D bioprinted tumor model and T cell infiltration

Stereolithographic printing of 3D models was previously described (18). To assess T cell infiltration, each 3D bioprinted tumor was overlaid (in medium) with 3 x 10^6^ T cells (either untransduced as control or L1CAM-CAR T cells utilizing CD28 or 4-1BB co-stimulation) at an effector to target ratio of 5:1 (E:T=5:1). T cells were allowed to infiltrate for 24 h. The 3D printed model was separated from the overlay medium containing T cells that had not infiltrated, and washed twice with PBS to exclude transfer of T cells on the 3D model surface. Each sample of T cell-infiltrated 3D printed models was enzymatically digested into a single-cell suspension as previously described (18), from which viable CD3^+^ cells were flow cytometrically sorted to obtain tumor-infiltrated CAR T cells. Viable non-infiltrated CD3^+^ T cells were also sorted from the overlay medium. RNA sequencing was applied to the sorted cell samples.

### 2.5 Cytokine release assays

For cytokine release assays, wells (48-well plates) were seeded with 0.15x10^6^ L1CAM-CAR T or control T cells (untransduced T cells, L1CAM-CAR T cells overexpressing only the GFP tag to control for enforced GFP-tagged SH2D2A expression) cells and stimulator cells (SK-N-BE (2) neuroblastoma cell line) at an effector:target ratio of 1:1. All data points were performed as technical triplicates. After 24 h, conditioned medium was collected from the cocultures and stored at –80°C until IFNG analysis using the OptEIA™ Set (cat#555142, BD Biosciences) ELISA kits according to the manufacturer’s instructions.

### 2.6 Flow cytometry

Cell surface expression of CD3 (clone Hit3a; Biolegend) and CD8 (clone SK1; Biolegend) were detected by fluorophore-conjugated monoclonal antibodies on a Fortessa X-20 (BD Biosciences, Franklin Lakes, NJ, USA) 4-laser flow cytometer. EGFRt expression was detected using biotinylated cetuximab (Bristol-Myers Squibb) and a phycoerythrin (PE)-conjugated streptavidin antibody (Biolegend). Activation was assessed by fluorophore-conjugated monoclonal antibodies detecting TNFRSF9 (formerly CD137, clone 4B4-1; Biolegend) and inhibitory receptor expression was assessed by using fluorophore-conjugated monoclonal antibodies detecting CD366 (also TIM3, clone F28-2E2, Biolegend), CD179 (also PD1, clone EH12.2H7; Biolegend), CD223 (also LAG3, clone 11C3C65, Biolegend). Dead cells were excluded from analyses using the LIVE/DEAD™ Fixable Green Dead Cell Stain Kit (Life Technologies). Flow cytometry data was processed using FlowJo V10 Software (Tree Star Inc., Ashland, OR, USA).

### 2.7 Cytotoxicity assay

T cell cytotoxicity was measured via live cell imaging on the IncuCyte platform (Sartorius, Goettingen, Germany, Models S3, SX5). Target cells were transduced with the mKate nucleic red fluorescent protein and were seeded 4 hours before adding L1CAM-CAR T cells at indicated effector-to-target ratios. Analysis was performed using the IncuCyte software (Sartorius, version 2022B).

### 2.8 Migration assays

Migration assays used polycarbonate transwell inserts with a 5-µm pore size fitted for 24-well plate wells. An endothelial cell layer was added by seeding 6x10^4^ HMEC-1 endothelial cells onto the top compartment of the transwell insert surface and labeled with VibrantTM DiO (cat# V22886, Thermo fisher scientific) overnight to enable visual cell detection. Confluency of endothelial cell layer was confirmed on the following day, before adding 5x10^5^ un-/modified T cells in 100 µl RPMI supplemented with 0.5% FCS to the top compartment, and adding 500 µl RPMI supplemented with 10% FCS and 100 ng/ml CXCL11 (SELPLG; cat# AF-300-46-20, Peprotec) or 100 ng/ml CXCL12 (SH2D2A; cat# 300-28A, Peprotec) chemoattractant to the bottom of the wells. Cell migration was imaged over 4 h using IncuCyte S3 instrument (Essen Bioscience). The IncuCyte 2021B software calculated the number of cells migrating through both the endothelial cell layer and membrane at each time point. The time point at which 50% of the T cells in a given experiment was determined using the time point at which no further migration occurred as 100% migration (M_max_) and the mean migrating cell number from sets of 3 consecutive measurements in the formula, M(t)=0.5×M_max_.

### 2.9 RNA sequencing and analysis

Bulk RNA was isolated from T cell samples using the Qiagen RNeasy Microkit. The RNA library was prepared using SMARTer^®^ Stranded Total RNA-Seq Kit v2 - Pico Input Mammalian Takara) following the manufacturer’s protocol. RNA Sequencing was performed using Hiseq4000. Sequencing reads were aligned with STAR 2.7.1a against the human genome reference GRCh38, and with bowtie2 against ribosomal RNA sequences to quantify rRNA contamination. Transcript models from the GENCODE v30 catalog were used to aid spliced alignments with STAR and to count mapped reads at the gene and exon level using featureCounts. Overall statistics on the alignment process were collected with FastQC and all feature Counts were then aggregated into a single Digital Gene Expression count-matrix and subjected to differential gene expression analysis using the DESeq2 R package. Transcription Factors were identified by membership in the GO: TF list included in the R package topGO, which was also used for all subsequent Gene Ontology enrichment analyses.

To perform HLA typing, mRNA sequencing reads were first mapped with BWA-MEM 0.7.17-r1188 against an index of human HLA genes to filter out relevant reads. These were then subjected to HLA-typing analysis using the optitype python package.

10X Genomics single cell sequencing, T cell samples were labeled with TotalSeq–C antibodies (Biolegend) according to manufacturer’s protocol, allowing to include all samples in a single sequencing run. T cell populations were flow cytometrically sorted for CD3^+^ cells. Libraries were prepared using Chromium Next GEM Single Cell 5’ Reagent Kits v2 (10X Genomics) according to manufacturer’s protocol without B and T cell receptor profiling. RNA Sequencing was performed using NovaSeq SPv1xp. Raw sequencing reads were processed with CellRanger according to official 10X protocol. The count matrix was further processed with Seurat using standard sc-RNAseq practice. Briefly, genes that were not expressed in at least one cell were discarded from the analysis, cells with less than 10,000 UMIs, less than 1,000 detected genes were discarded (cutoff chosen based on the distributions to separate encapsulated cells from background). Highly variable genes identified using nodelGeneCV2. Counts were then normalized using logNormCounts.

### 2.10 Differential gene expression analysis of public dataset

Publicly available single-cell RNA-sequencing data from Zheng et al. (23) was processed to examine SH2D2A expression across tumor types. Raw count matrices and metadata were obtained from GEO (GSE156728) and processed with Seurat using standard single-cell RNA-seq workflows. Cells with fewer than 5,000 detected genes or greater than 10% mitochondrial gene content were excluded. Counts were log-normalized, and differential gene expression between tumor and healthy cells was computed using the FindMarkers function, restricting analysis to genes expressed in at least 25% of cells in either group and with a minimum absolute log fold change of 0.25.

### 2.11 Statistical analysis

Student’s T-test was used for two-factor comparisons to evaluate the mean values performed in GraphPad Prism 9 (GraphPad Software). All experiments were independently repeated (n = 3-5). Data are presented as mean ± SD and all graphs were generated and analyzed using GraphPad Prism 9. Expression of migration-related genes in distinct 3D-model-infiltrating T cell subgroups from the 10X single-cell RNA sequencing data were compared using a Kruskal-Wallis test. Statistical significance was defined in all tests as p < 0.05.

## 3 Results

### 3.1 RNA bulk sequencing identifies migration-related gene expression profiles of CAR T cells

**Figure 1:**
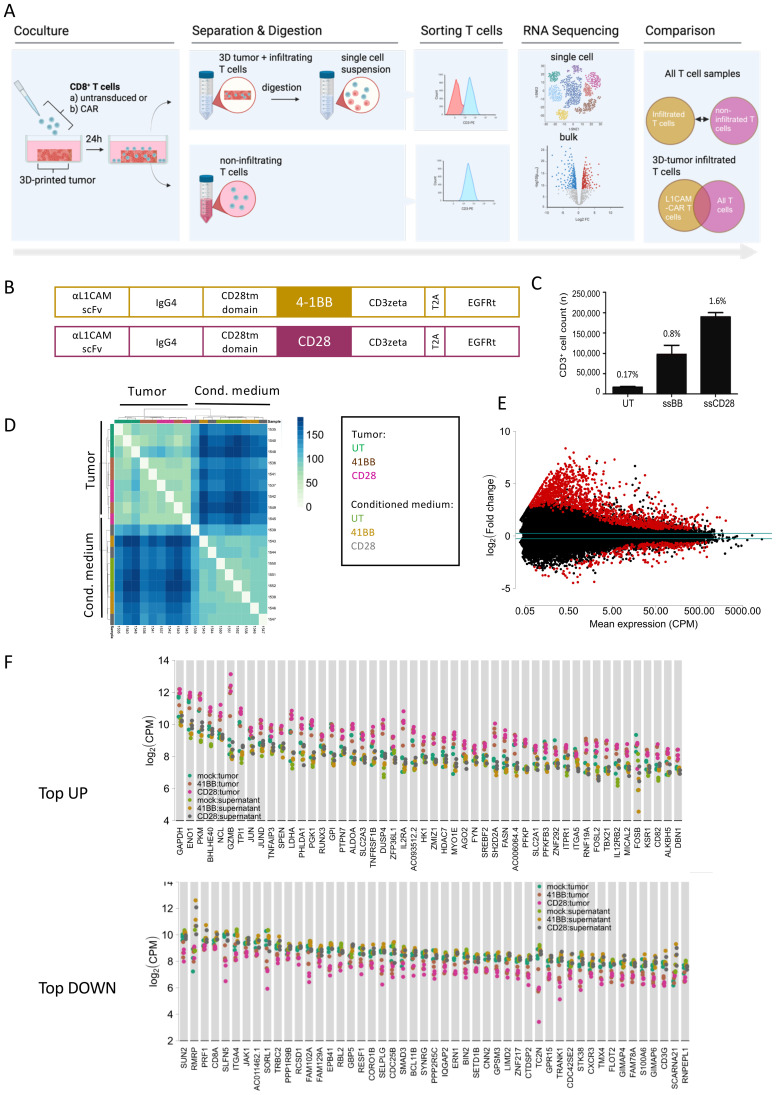
Bioprinted 3D tumor model-infiltrating T cells show distinct genetic features compared to non-infiltrating T cells. **(A)** Schematic of experimental set up. L1CAM-CAR T or untransduced (UT) T cells were cocultured for 24 h with a 3D bioprinted neuroblastoma model. Non-infiltrated T cells and 3D bioprinted tumors were separated and enzymatically digested into single cell suspension before cells from each sample were sorted for CD3^+^ cells. Sorted cells were then used for RNA bulk and 10X single cell sequencing. Differential gene expression between infiltrated and non-infiltrated T cells was analyzed for L1CAM-CAR T cells and untransduced T cells separately. Common differentially expressed genes of 3D-tumor infiltrated L1CAM-CAR T cells and untransduced T cells were analyzed for genes regulating cell migration. **(B)** Schematic of L1CAM-CAR constructs used. **(C)** Number of T cells isolated from the 3D bioprinted neuroblastoma model after 24 h of coculture. n=4, mean ± SD. The values shown represent the absolute proportion of infiltrated T cells relative to the total number of T cells seeded. **(D)** Heat map of 3D tumor model- infiltrated and non-infiltrated (CAR) T cells indicating up- (blue) and downregulated (green) genes. **(E)** Volcano plot of differentially expressed genes between all 3D-tumor infiltrated T cells (L1CAM-CAR + untransduced T cells) and non-infiltrated T cells. **(F)** Top 50 up- and downregulated genes in differentially expressed genes comparing all samples from infiltrated versus non-infiltrated (CAR) T cells. CPM, count per million; UT, untransduced T cells; CD28, CD28-costimulated L1CAM-CAR T cells; 4-1BB, 4-1BB costimulated L1CAM-CAR T cells.

**Figure 2:**
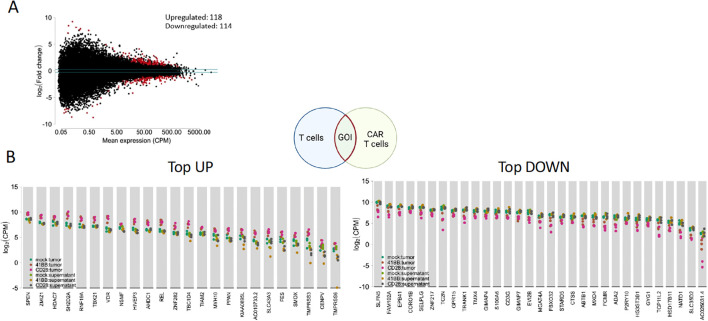
**(A)** Volcano plot of differentially expressed genes between all 3D-tumor infiltrated CAR T cells (CD28 and 4-1BB costimulated L1CAM-CAR T cells) and non-infiltrated CAR T cells. **(B)** Schematic of the meta-analysis approach filtering the genes of interest (GOI) that overlap in differentially expressed genes in infiltrated L1CAM-CAR T cells and untransduced T cells. Upregulated and downregulated genes found in meta-analysis. CPM, count per million.

We employed our previously established 3D bioprinted neuroblastoma model (18) as a substitute for the solid tumor matrix to challenge CAR T cell migration and separate T cells into groups capable or incapable of infiltration (**Figure 1A**). The 3D-tumor models were cocultured with CD28- or 4-1BB-costimulated L1CAM-CAR T or untransduced T cells. T cells were present for 24 h before being isolated from the tumor (infiltrating T cells) or the conditioned medium (non-infiltrating T cells) (**Figure 1B**). We hypothesized that RNA profiling in the two groups (infiltrating versus non-infiltrating T cells) should identify genes or signaling networks essential for tumor infiltration. The different costimulatory domains impacted L1CAM-CAR T cell infiltration. Nearly twice as many CD28-costimulated L1CAM-CAR T cells infiltrated (mean=196,839 T cells, range=190,276-201,389 cells) the tumor model compared to 4-1BB costimulated L1CAM-CAR T cells (mean=104,381 T cells, range: 87,304-129,403; **Figure 1C**). Untransduced T cells were able to migrate into the tumor model (mean=22,334 cells, range=20,203-23,004 cells), but their numbers corresponded to only about 10% of the CD28-costimulated and 25% of the 4-1BB–costimulated L1CAM-CAR T cells that infiltrated the 3D tumor model (**Figure 1C**). We speculate that control cells entered the tumor model because gravity-assisted cell interactions, and not active migration, since T cells were added on top of the tumor model. We hypothesized that CAR T cell antigen recognition may induce migratory pathways enabling superior CAR T cell infiltration (compared to unactivated controls) and different costimulatory domains may drive migration through distinct mechanisms, altering infiltrative abilities. We applied bulk RNA sequencing to both populations (infiltrating and non-infiltrating) to identify genes and potential signaling pathways that either commonly or differentially regulate T cell migration. Bulk RNA sequencing data underwent filtering for stringent quality control, excluding 60% of reads in comparative analyses, due to low sample RNA concentrations. Only samples with at least 2.0 x 10^7^ mapped reads were used in analyses. Biological triplicates for each experiment sample clustered closely together (principal component analysis), excepting 2 outlier samples (sample 1542: infiltrating 4-1BB-costimulated L1CAM-CAR T cells, sample 1442: non-infiltrating CD28-costimulated L1CAM-CAR T cells; **Supplementary Figure 1A**). This quality assessment indicates experiments replicated the event being tested well. Gene expression patterns in tumor-infiltrating T cells were distinct from non-infiltrating cells (unsupervised clustering, all samples; **Figure 1D**), indicating regulatory mechanisms may be different between T cell populations. Distinct gene expression patterns were differentially upregulated (1,390 genes) and downregulated (287 genes) in any T cell group infiltrating the tumor model (**Figure 1E**; FDR-adjusted p-value <0.1, fold-change I >1.2I), when the 3 T cell populations entering the tumor model were compared (differential gene expression analysis) to all 3 T cell populations (pooled) not entering the tumor model (**Figures 1E, F**). Gene sets related to hypoxia (gene set enrichment analysis; **Supplementary Figure 1B**), glycose metabolism and cytokine-mediated signaling (gene set enrichment analysis; **Supplementary Figure 1C**) were strongly upregulated in infiltrating (CAR) T cells. Differential gene expression suggests the infiltration processes may utilize multiple signaling-distinct mechanisms. To better focus on mechanisms used by each set of infiltrating L1CAM-CAR T cells, differential gene expression analysis was applied to samples paired according to costimulatory mechanism and ability to infiltrate. We hypothesized that this analysis could delineate discrete regulatory pathways or gene expression used by migrating L1CAM-CAR T cells. Infiltrating (CAR) T cells differentially upregulated 118 and downregulated 116 genes compared to (CAR) T cells incapable of tumor infiltration (FDR-adjusted p-value <0.1 fold, change I>1.2I; **Figure 2A**). Gene expression profiles in infiltrating L1CAM-CAR T cells differed somewhat for the costimulation method used (CD28 vs. 4-1BB; 38 genes upregulated, 50 genes downregulated; FDR-adjusted p-value <0.1, fold change I>1.2I; **Supplementary Figure 1D**). Costimulating domain may impact migration mechanisms used to enter our 3D tumor model, at least in the first 24h. Given that infiltrated L1CAM-CAR T cells recognized the antigen at tumor encounter, gene expression levels may be influenced by antigen-dependent CAR T cell activation. To mitigate this bias, a meta-analysis was conducted seeking differentially expressed genes using the initial analysis for all infiltrating T cell groups against all T cell groups (pooled) that did not enter the tumor model with the second CAR-specific analysis (**Figure 2B**). This approach aimed to exclude genes regulated by T cell activation (by including the untransduced control cells), while identifying genes driving (CAR) T cell migration. Five genes functionally involved in cell migration (upregulated: SH2D2A, TIAM2, MYH10; downregulated: CORO1B, SELPLG; **Table 1**) were differentially regulated among the total 24 upregulated and 32 downregulated genes (**Figure 2B**). Bulk RNA sequencing from T cell groups capable or incapable of infiltrating the 3D bioprinted neuroblastoma models revealed that infiltrating T cells equipped with either CAR (or lacking a CAR) differentially expressed migration-associated genes and strongly upregulated gene sets related to glycose metabolism, cytokine-mediated signaling and hypoxia.

**Table 1:** Migration-associated genes found in infiltrating (CAR) T cells based on RNA-sequencing analysis.

Gene (Encoded protein)	Function	References
Upregulated		
SH2D2A (T cell-specific adapter protein, SH2D2A)	Essential for Chemokine-mediated signaling events that lead to T cell migration. Promotes migration by CXCL12-induced actin polymerization.	(31, 36)
MYH10 (Non-muscle myosin II B)	Non-muscle myosin II B (NM IIB) is a member of the motor protein family that generate forces for front-back cell polarity of migrating cells by crosslinking and contracting actin, promoting linear structures of bundled filaments.	(50, 51)
TIAM2/STEF (T cell lymphoma invasion and metastasis 2)	Encodes a guanin nucleotide exchange factor that activates Rac1 and regulates invasion-related genes in cancer. Plays an important role in regulating the perinuclear actin cap responsible for nuclear morphology and re-orientation during front-back cell polarity. Co-localizes with NM IIB at the perinuclear actin cap to regulate contractile forces.	(52–54)
Downregulated		
CORO1B (Coronin 1b)	Actin-binding protein known to regulate cytoskeletal dynamics at the leading edge during cell migration.	(55–58)
SELPLG (SELPLG, P-selectin glycoprotein ligand-1, PSGL-1),	Adhesion receptor that binds to P-, L-, and E-selectin and mediates cell adhesion to endothelium, platelets and in between cells.	(38, 59, 60)

### 3.2 Single-cell sequencing identifies SH2D2A and SELPLG as candidate genetic regulators of T cell migration

**Figure 3:**
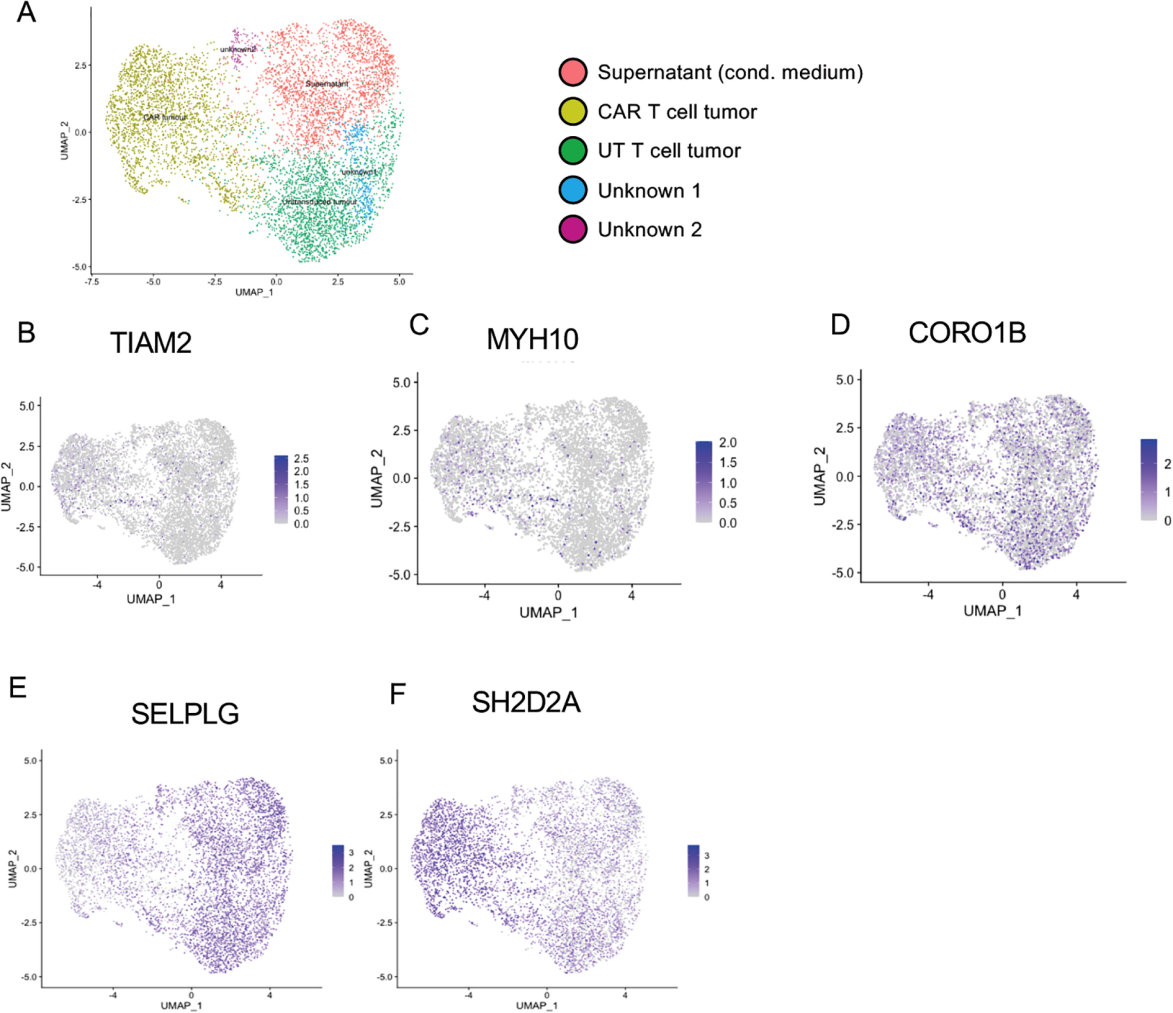
Single cell RNA sequencing verifies SH2D2A and SELPLG as candidate genes to regulate CAR T cell migration. **(A)** UMAP embedding and unsupervised cell clustering based on 10X genomics scRNA sequencing data of the pooled T cell library following 24 h coculture with 3D neuroblastoma model. Shown are data from 8,964 T cells clustering into 5 clusters, including L1CAM-CAR T cells (2,228 cells) and untransduced T cells (2,092) isolated from the tumor as well as non-infiltrated CAR and untransduced T cells (together 2,251). TIAM2 **(B)**, MYH10 **(C)**, CORO1B **(D)**, SELPLG **(E)** and SH2D2A **(F)** comparing 3D tumor-model infiltrating and non-infiltrated T cells. Darker colors in the color scale bar indicate stronger gene expression. Grey indicates an absence of detectable expression.

To reinforce and validate results obtained from bulk RNA sequencing analysis prior to sequencing, we used TotalSeq antibodies to label each sample with a unique oligonucleotide barcode, enabling reliable de-multiplexing and cell assignment after sequencing. We conducted single-cell RNA sequencing on the best infiltrating L1CAM-CAR T cells (CD28-costimulated) and those L1CAM-CAR T cells that did not enter the 3D bioprinted model (plus untransduced T cell controls) in a new experiment. Since bulk RNA sequencing assumes uniformity across cell populations, cell heterogeneity would be masked and rare cell profiles would not be identified. This may have been the reason behind only minor differences among T cell groups being identified by differential gene expression analysis from bulk RNA sequencing data (**Supplementary Figure 1B**). This experiment yielded RNA sequenced (mean: 28,709 reads/cell) from a total of 8,964 cells (following preprocessing with Cell Ranger) including 4,320 infiltrating and 2,251 not infiltrating cells. Unsupervised clustering of the single cells that were profiled identified 5 different clusters (**Figure 3A**). Non-infiltrating T cells clustered into their own group, regardless of transduction, but infiltrating T cells clustered into groups transduced with the L1CAM-CAR and untransduced. Two further clusters could not be assigned to a functional group when transcriptional information and the labeling antibodies were integrated. This clustering supported the idea that L1CAM-CAR T cells used separate mechanisms to enter the tumor model compared to untransduced control T cells, and that non-infiltrating T cells may not be entering the tumor model for common reasons. The tumor-infiltrating T cell groups (LICAM-CAR T and untransduced T cells) more strongly expressed migration-associated gene sets related to lymphocyte migration (**Supplementary Figure 2A**) and regulation of lymphocyte migration (**Supplementary Figure 2B**) as analyzed in gene set enrichment analysis. These findings indicate active processes, although potentially different ones in CAR-transduced and control T cells, are driving tumor model entry and migration. To investigate whether the 5 candidate genes (*MYH10*, *TIAM2*, *CORO1B*, *SH2D2A*, *SELPLG*) identified by bulk RNA sequencing were also among the differentially regulated genes in single infiltrating (CAR) T cells that independently clustered compared to the pooled (CAR) T cell groups incapable of tumor model entry, we overlaid *MYH10*, *TIAM2*, *CORO1B* and *SH2D2A* expression levels and *SELPLG* onto the UMAP visualization (**Figures 3B–F**). To assess unique features of 3D-tumor infiltrating T cells, we compared expression levels of the 5 genes in UMAP clusters that corresponded to 3D-tumor infiltrating (green and yellow clusters) or non-infiltrating (red cluster) T cells (UMAP unsupervised clustering, **Figure 3A**). Only a limited number of T cells expressed MYH10 and TIAM2, but expression was not confined to any particular cluster (**Figures 3B, C**). CORO1B expression was higher than for MYH10 and TIAM2, but was also not specific for any cluster (**Figure 3D**). SH2D2A was specifically upregulated in infiltrating T cells (2.24-fold change, adjusted p = 1.09e-192; **Figure 3E**), while SELPLG was specifically downregulated (-2.19-fold change, adjusted p = 5.918613e-229; **Figure 3F**) in infiltrating T cells. Differential gene expression was stronger in the L1CAM-CAR T cell population than the untransduced controls (**Figures 3E, F**). Infiltrating and non-infiltrating T cells (pooled untransduced T cells and L1ACM-CAR T cells) were sub-divided into SELPLG-positive and SELPLG-negative or SH2D2A-positive and SH2D2A-negative cell groups. Expression analysis of key regulators of cell migration (CXCR3, CCR7, CXCR5 and ITGAL, which encodes LFA1, an alias protein name) within these groups revealed significant upregulation of CCR7 in a subset of tumor-infiltrating SH2D2A-positive T cells and CXCR3 in infiltrating SH2D2A-positive T cells (**Supplementary Figure 2C**). In contrast, no difference in CXCR5 expression was detected between these samples and ITGAL was less expressed in infiltrating SH2D2A-positive T cells. No differences in gene expression were observed in non-infiltrating T cells. These findings indicate that SH2D2A expression may enhance T cell migration by upregulating receptors such as CCR7 and CXCR3. In SELPLG-deficient tumor-infiltrating T cells, expression analysis of the same selected genes revealed that CCR7 and ITGAL were significantly upregulated in SELPLG-negative cells that infiltrated the tumor model (**Supplementary Figure 2D**). In contrast, CXCR3 was less expressed in SELPLG-deficient infiltrating T cells and CXCR5 expression was similar across the samples (**Supplementary Figure 2D**). CCR7 binds to the chemokines CCL19 and CCL2, functionally directing T cells to and within the lymph nodes (24) but also enhancing velocity of T cell migration (25). ITGAL binds to the Intercellular adhesion molecule family (ICAM) facilitating firm adhesion of leukocytes to the endothelium (26). The upregulation of these receptors may suggest that SELPLG-deficient T cells rely more heavily on these molecules for migration, although this cannot be directly linked based on the current analysis. Taken together, our single-cell RNA expression analysis affirmed that SELPLG and SH2D2A are differentially regulated in single tumor-infiltrating L1CAM-CAR T cells, suggesting a functional role, while TIAM2, CORO1B and MYH10 do not seem to influence L1CAM-CAR T cell migration.

### 3.3 L1CAM-CAR T cell *in vitro* migratory capacity unchanged by overexpressing SH2D2A

To strengthen our findings from single-cell RNA profiling, we re-analyzed publicly available datasets. Zheng et al. previously compared tumor-infiltrating T cells to T cells circulating in blood samples collected at surgery from the same patients in 316 patients being treated for 21 solid tumor entities (23). We analyzed whether SH2D2A was also upregulated in tumor-infiltrating T cells in this dataset. SH2D2A was upregulated in T cells that infiltrated ovarian and renal cancers and multiple myeloma, but was downregulated in T cells infiltrating breast cancers (**Table 2**), supporting an inverse mechanistic role for SH2D2A dependent on tumor type.

**Table 2:** Differentially expression of SH2D2A in tumor-infiltrated T cells vs. T cells found in the blood from the same patient from 21 different solid tumor entities (23).

Tumor entity	avg_log2FC	p_val	pct.1	pct.2	p_val_adj
Ovarialcarcinoma	0.425	3,74E-21	0.898	0.711	9,0216E-17
Renal carcinoma	0.305	1,03E-58	0.732	0.623	2,48E-54
Breast cancer	-0.486	5,91E-28	0.570	0.696	1,43E-23
Esophageal carcinoma	-0.254	4,34E-24	0.622	0.671	1,05E-19
Multiple myeloma	0.717	3,10E-96	0.672	0.426	8,94E-92
Uterine corpus endometrial carcinoma	-0.275	2,68E-52	0.711	0.748	6,47E-48

We analyzed if SH2D2A is also down- or upregulated in tumor-infiltrated T cells compared to T cells isolated from blood of the same patient. Data sets from tumor entities are depicted in which SH2D2A was significantly differentially expressed.

**Figure 4:**
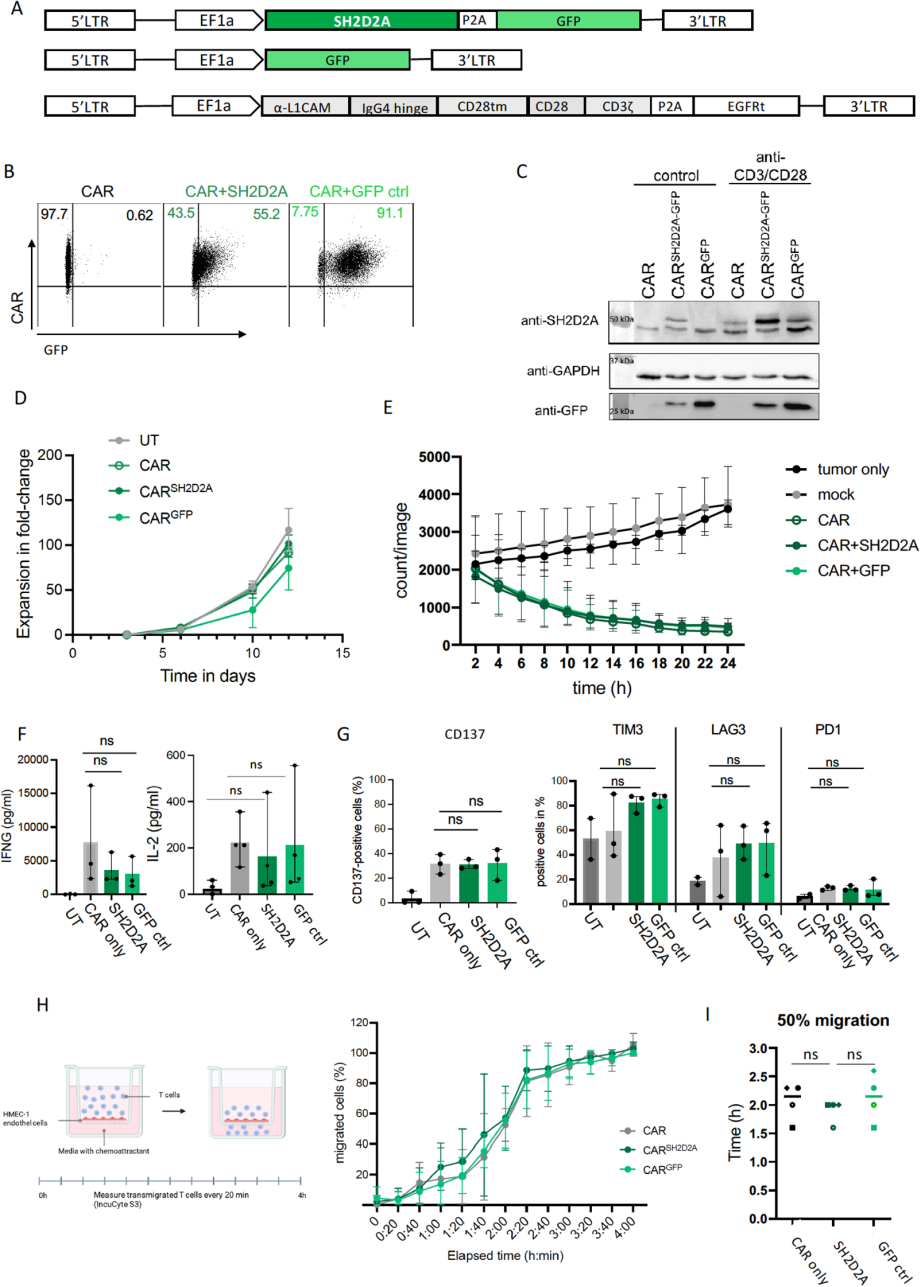
Overexpression of SH2D2A does not affect L1CAM-CAR T cell efficacy but fails to improve migratory capacity. **(A)** Schematic of constructs for SH2D2A overexpression with GFP transduction marker (top), GFP control construct (middle) and the L1CAM-CAR construct (bottom). LTR, long terminal repeat; TM, transmembrane domain. **(B)** Dot plot of flow cytometry analysis of transduction efficacy for CAR^+^/GFP^+^ cells upon enrichment. Gating was applied for living CD3^+^ cells. **(C)** Western blot confirms SH2D2A overexpression in untreated T cells (control) and after 24 h anti-CD3/CD28 stimulation using anti-SH2D2A and anti-GFP antibody. GAPDH served as loading control. **(D)** Line graph showing expansion of different L1CAM-CAR T cells over 12 days. **(E)** Cytotoxicity of different L1CAM-CAR T cells against SK-N-BE (2) neuroblastoma cells over 24 h in an E:T-ratio of 5:1. IFNG release after 24 h coculture with SK-N-BE (2) cells. n=3, mean ± SD. **(F, G)** IFNG and IL2 release **(F)** and cell surface expression if CD137 activation marker and inhibitory receptors **(G)** after coculture in an E:T of 1:1. N = 3, ns= not significant unpaired t-test. **(H)** Schematic of the transendothelial migration assay. HMEC-1 endothelial cells were cultured on a transwell insert. T cells were added to the upper chamber and allowed to migrate through the endothelial barrier towards a CXCL12 chemoattractant gradient in the lower chamber. Line graph showing percentage of migrated cells for the different T cell subtypes over time. **(I)** Calculated time points of 50% migration for the different T cell subtypes. n=4 for two different T cell donors. Symbols indicate the individual biological replicates.

We aimed to functionally validate how SH2D2A impacts CAR T cell migratory capacity using a gene modification approach. SH2D2A encodes a T cell-specific adapter protein (TSAd) that orchestrates several TCR-initiated cytoplasmic signaling pathways. We overexpressed SH2D2A in L1CAM-CAR T cells using an SH2D2A expression construct inserted upstream of the P2A-separated green fluorescent protein (GFP, serving as the transduction marker) that was driven by the EF1α promoter in the epHIV7 lentiviral vector (**Figure 4A**, lentiviral vector expressing only GFP served as control). The CD28 costimulated L1CAM-CAR was expressed via a second lentiviral vector that co-expressed a P2A-separated truncated EGFR (EGFRt) as a transduction marker. Reporter gene expression verified cotransduction of the L1CAM-CAR and SH2D2A construct in 55.2% of L1CAM-CAR T cells and co-transduction of the GFP control plasmid and the L1CAM-CAR in 91.1% of L1CAM-CAR T cells. L1CAM-CAR expression was verified in 98% of single transduced T cells (**Figure 4B**). SH2D2A overexpression was validated on the protein level in western blotting (**Figure 4C**). Since TCR-activation induces protein translation, we also assessed SH2D2A expression in all T cell subtypes following 24-hour anti-CD3/CD28 stimulation. SH2D2A and GFP protein expression markedly increased in the activated SH2D2A-overexpressing L1CAM-CAR T cells and to some extent also the untransduced control T cells (**Figure 4C**, full western blot image in supplementary **Figure 3A**). An unspecific protein signal below 50 kDa was detected in all samples, consistent with previous observations (27). Proliferation capacity was comparable across all T cell groups, demonstrating that SH2D2A overexpression does not alter CAR T cell viability or proliferative capacity (**Figure 4D**). Cytotoxicity to SK-N-BE (2) neuroblastoma cells (challenged 24h in coculture) was comparable among L1CAM-CAR T cells, L1CAM-CAR T cells co-transduced with GFP-tagged SH2D2A or co-transduced with GFP alone (mean lysis: L1CAM-CAR=77.18%, L1CAM-CAR co-transduced with GFP-tagged SH2D2A = 66.3%, L1CAM-CAR cotransduced with GFP = 68.19%; **Figure 4E**). Our findings verify that overexpressing SH2D2A did not affect L1CAM-CAR T cell cytolytic activity *in vitro*. Likewise, SH2D2A overexpression did not alter T cell activation, since IFNG and IL2 release was unaffected in cocultures (equal among the L1CAM-CAR T cell subtypes, untransduced T cells did not release IFNG or IL2; **Figure 4F**). The CD137 activation marker was also equally expressed in all L1CAM-CAR T cell groups (mean % of cells in group expressing CD137: L1CAM-CAR=31.71%, L1CAM-CAR cotransduced with GFP-tagged SH2D2A = 28.99%, L1CAM-CAR cotransduced with GFP = 28.76%; **Figure 4G**). Fewer than 2% of untransduced T cells expressed CD137 in these experiments (mean CD137^+^ cells= 1.32%: **Figure 4G**). The TIM3, LAG3 and PD-1 inhibitory receptors were also equally expressed in the three L1CAM-CAR T cell groups (**Figure 4G**). Collectively, our data imply that overexpressing SH2D2A does not interfere with L1CAM-CAR T cell mechanisms used to recognize and kill tumor cells.

Migratory capacity of all T cell groups was assessed in transendothelial-transwell *in vitro* assays. To model an endothelial barrier, a monolayer of the HMEC-1 endothelial cell line was plated onto the uncoated transwell insert, and T cells were added to the upper chamber. Migration along a chemotactic gradient to CXCL12 added to the lower well was then assessed by quantifying cells that migrated into the lower chamber. Unexpectedly, all L1CAM-CAR T cell groups (CAR alone, CAR+GFP, CAR+ GFP-tagged SH2D2A) migrated with similar dynamics, reaching an end to active migration at 3 h (**Figure 4H**). The L1CAM-CAR T cells co-expressing GFP-tagged SH2D2A migrated the fastest, with 50% of cells migrating at 2h (**Figure 4I**). The other T cell groups harboring the L1CAM-CAR followed with only the slightly longer migration velocity of 2h 9min (L1CAM-CAR alone, L1CAM-CAR+GFP; **Figure 4I**). Our data indicate only a minor effect of SH2D2A overexpression on T cell migration in this experimental setting. Since 34.8% of the population of L1CAM-CAR T cells co-transduced with GFP-tagged SH2D2A did not contain the GFP-tagged SH2D2A construct, we hypothesized that SH2D2A-overexpressing cells might be selectively enriched within the migrating cell population. Contrary to expectations, no enrichment with SH2D2A-overexpressing cells occurred in the migrating population at any point during the experiment (flow cytometric analysis of GFP-signal in T cells before experiment intitation and in migrating T cells after 2 and 4 h, **Supplementary Figure 3B**). This further indication that SH2D2A overexpression did not improve migration made us look specifically at CXCR4/CXCL12-mediated T cell migration, in which SH2D2A is known to be involved. We hypothesized that overexpressing SH2D2A should also upregulate CXCR4 on the L1CAM-CAR T cell surface. However, CXCR4 was equally expressed on L1CAM-CART cells with or without GFP-tagged SH2D2A (50.6% and 54.1% CXCR4-positive cells, respectively; **Supplementary Figure 3C**). CXCR4 was downregulated 6.5-fold from initiation to the end of the experiment in L1CAM-CAR T cells overexpressing SH2D2A. Similar, but less pronounced, CXCR4 expression was regulated in L1CAM-CAR T cells expressing endogenous SH2D2A levels. CXCR4 expression decreased 3.3-fold from the start to the end of the experiment (CXCR4 downregulation: 1.3-fold at 2 h and 3.3-fold at 4 h; (flow cytometric analysis of CXCR4 surface expression in T cells before experiment initiation and in migrating T cells after 2 and 4 h, **Supplementary Figure 3B**). These results suggest that at least in this experimental setting, L1CAM-CAR T cells do not exploit the CXCR4/CXCL12-mediated T cell migration axis. Our *in vitro* findings support that SH2D2A does not improve or significantly alter the invasive or migratory capacity of L1CAM-CAR T cells.

Because SH2D2A (alias, TSAd protein) also plays a crucial role in T cell signaling by interacting with signaling proteins, such as LCK, a SRC family tyrosine kinase that is central to initiating TCR signaling (28, 29), we investigated the possibility that SH2D2A may be used as CAR T cell activation marker by comparing expression belonging to single cells (RNA sequencing). Gene sets related to T cell activation were more strongly expressed in *SH2D2A*-expressing T cells (compared to *SH2D2A*-negative cells) that infiltrated the 3D-tumor model (**Supplementary Figure 3D**). Gene sets associated with T cell exhaustion were also upregulated in 3D-tumor infiltrating *SH2D2A*-expressing cells (**Supplementary Figure 3D**). These findings provide evidence that *SH2D2A* upregulation, resulting in increased TSAd protein promotes T cell activation, that likely accelerates T cell signaling and exhaustion. These data suggest that SH2D2A could also be used as CAR T cell activation marker.

### 3.4 SELPLG downregulation enhanced L1CAM-CAR T cell migration *in vitro* but not *in vivo*

**Figure 5:**
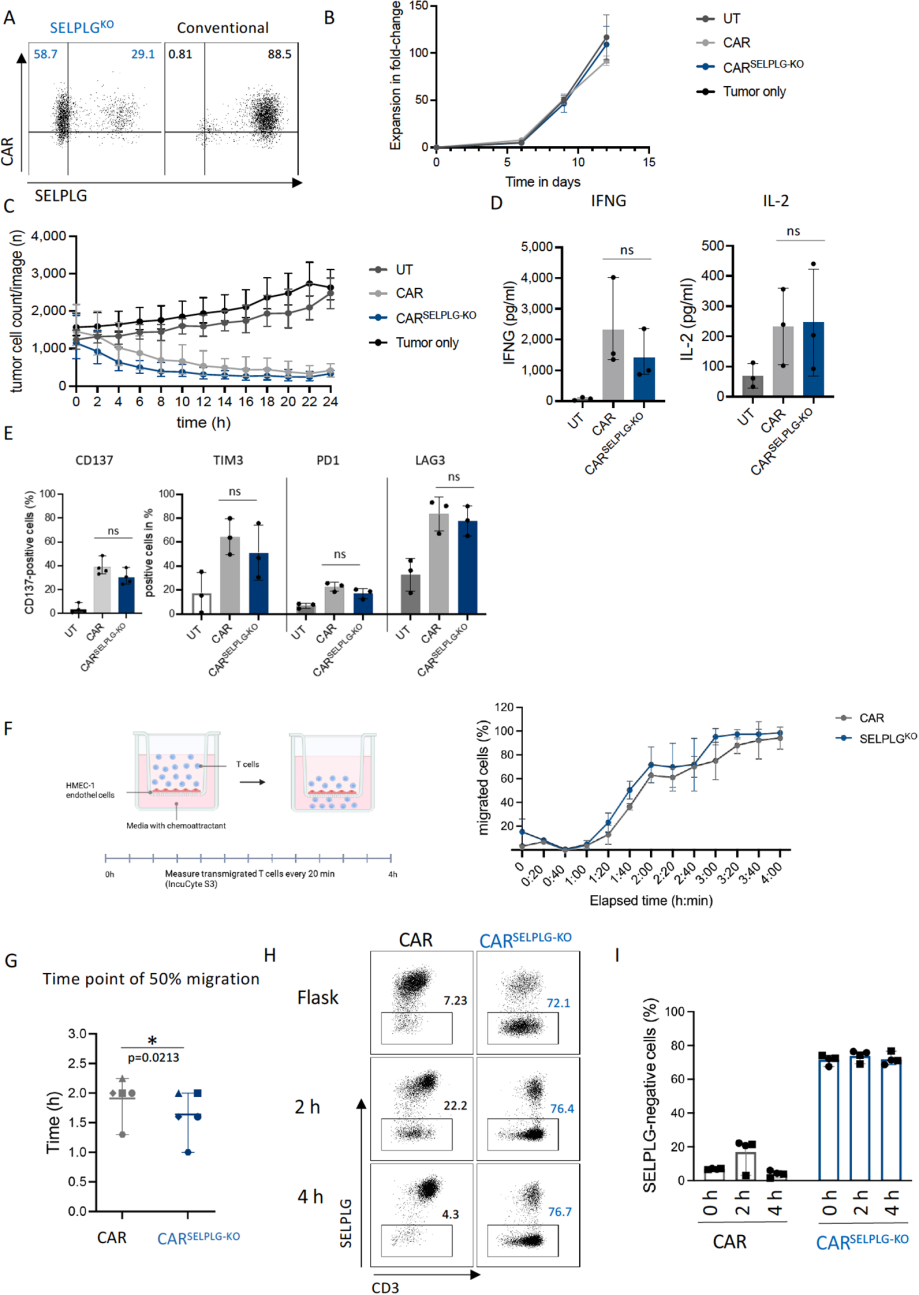
SELPLG-deficiency improves migratory capacity of L1CAM-CAR T cells *in vitro*. **(A)** Flow cytometry analysis of CAR transduction and SELPLG-knockout (also known as PSGL-1) efficacy after enrichment for EGFRt-positive cells. **(B)** Expansion in fold-change is shown for untransduced, CAR and CAR^SELPLG-KO^ T cells upon stimulation. Viable cells were counted manually using trypan blue. n=3, mean ± SD. **(C)** Tumor cell count over time in a coculture experiment with L1CAM-CAR T cells or untransduced T cells (UT). E:T=5:1, n=4, mean ± range. **(D)** IFNG and IL-2 release after 24 h coculture with SK-N-BE (2) cells. **(E)** Cell surface expression of CD137 activation marker and inhibitory receptors in L1CAM-CAR T cells or UT T cells after 24 h coculture (E:T=1:1; n=3, mean ± SD, unpaired t-test, ns= not significant) **(F)** Schematic of the transendothelial migration assay. HMEC-1 endothelial cells were cultured on a transwell insert. T cells were added to the upper chamber and allowed to migrate through the endothelial barrier towards a CXCL11 chemoattractant gradient in the lower chamber.T cell migration over time in transendothelial migration assay shown for different T cell subtypes. **(G)** Mean time point of 50% migration shown for two different donors. Symbols indicate individual replicated experiments **(I)**. (n=5, mean ± range; paired t-test, *p<0.05, effect size = mean difference = 0.27). **(H)** Exemplary dot plot and summary of flow cytometry analysis of SELPLG cell surface expression before (flask) start of experiments as well as depicted in a bar graph for indicated time points during the experiment (n=5).

Since our single-cell data indicated that SELPLG expression was reduced in migrating cells, we functionally analyzed how knocking out SELPLG would influence L1CAM-CAR T cell migration. SELPLG encodes for the glycoprotein cell adhesion ligand, selectin P (also known as PSGL-1), which is one of the high-affinity cell adhesion receptor molecules (P, E and L selectins) on activated endothelial cells (30). T cell rolling, the first step in transendothelial migration, is facilitated by T cell engagement with P selectin on activated endothelium at sites of inflammation. We generated T cells lentivirally transduced with a L1CAM-CAR (CD28-costimlated, co-expressing EGFRt transduction marker) together with guide RNAs targeting SELPLG for CRISPR/Cas9 knockout. We obtained 60% SELPLG-deficient L1CAM-CAR T cells, while 30% of L1CAM-CAR T cells in the population retained SELPLG expression (**Figure 5A**). In the control L1CAM-CAR T cell population retaining endogenous SELPLG expression, 88% of cells expressed the CAR (**Figure 5A**). Untransduced T cells were also used as a control for nonspecific T cell activity. SELPLG expression levels in untransduced T cells were similar to L1CAM-CAR T cells (supplementary **Figure 4A**), indicating that the presence of the CAR construct does not affect SELPLG cell surface expression. SELPLG deficiency did not affect T cell expansion over the 12-day time course (**Figure 5B**). Cytotoxicity against SK-N-BE (2) neuroblastoma cells (challenged 24h in coculture) was comparable between L1CAM-CAR T cells with and without SELPLG deficiency (**Figure 5C**). As expected, untransduced T cells did not kill tumor cells (**Figure 5C**). Similarly, IFNG or IL2 release were comparable between L1CAM-CAR T cells with or without SELPLG-knockout (**Figure 5D**). Expression levels of the CD137 activation marker or the TIM-3, PD-1 and LAG3 inhibitory receptors were also comparable in L1CAM-CAR T cells with or without SELPLG (all shown in **Figure 5E**). We conclude that SELPLG knockout did not alter any L1CAM-CAR T cell function, including T cell proliferation, survival, activation, cytolytic activity, effector cytokine production or exhaustion, *in vitro*.

Migratory capacity was assessed via *in vitro* transendothelial-transfilter migration assays (**Figure 5F**). SELPLG-deficient L1CAM-CAR T cells migrated faster than L1CAM-CAR T cells, achieving a mean difference of 24 minutes in the 4-hour *in vitro* migration assay (**Figure 5G**). The mean time at which 50% of the cell population without SELPLG had migrated was reached after 1 h and 36 min in comparison to 2 h for L1CAM-CAR T retaining endogenous SELPLG expression (**Figure 5G**). We hypothesized that SELPLG cell surface expression would be downregulated in the control L1CAM-CAR T cells during active migration, since our findings indicate that loss of SELPLG improves migratory capacity in L1CAM-CAR T cells. We confirmed this hypothesis using flow cytometric analysis of SELPLG expression on the control L1CAM-CAR T cell population. The fraction of SELPLG-negative control L1CAM-CAR T cells increased 3-fold within the first 2 hours (compared to cells from initial culture flask, reflecting the starting condition; **Figures 5H, I**). Our findings verified that SELPLG knockout significantly improved L1CAM-CAR T cell migratory capacity *in vitro*, without altering anti-tumor function, and demonstrated that CAR T cells dynamically downregulate SELPLG surface expression during migration.

**Figure 6:**
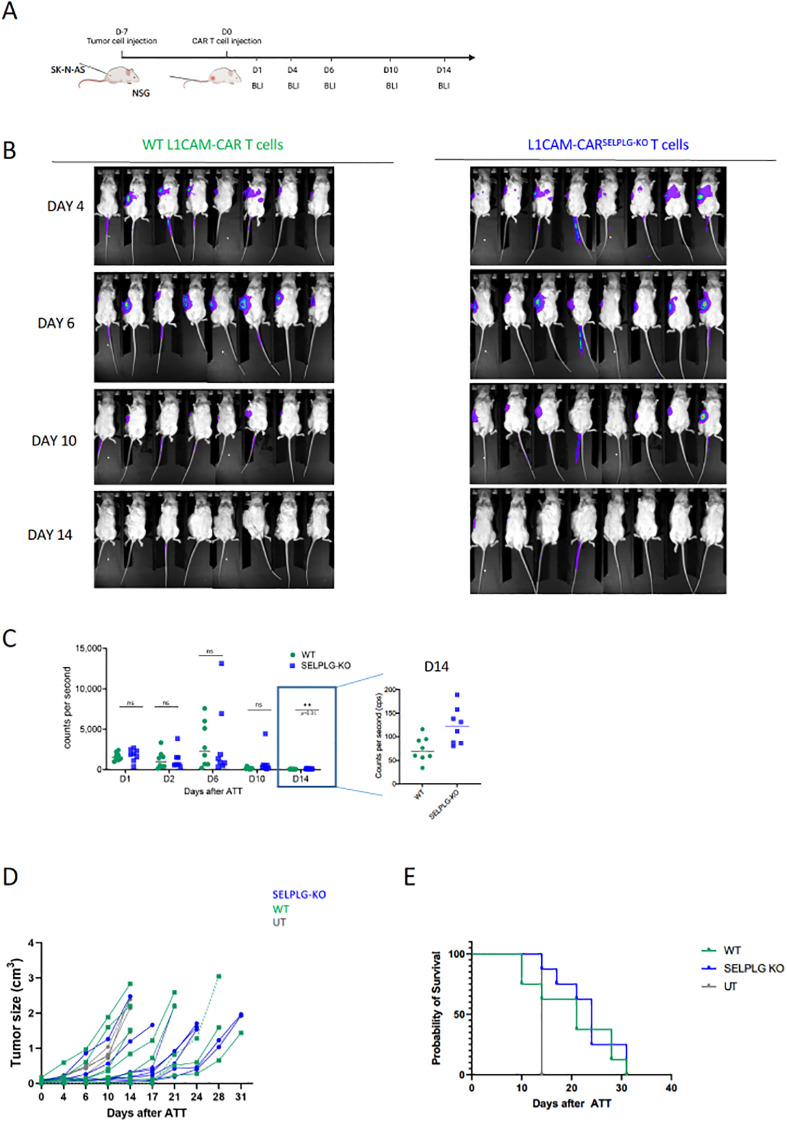
SELPLG-deficiency does not improve tumor infiltration and anti-tumor efficacy of L1CAM-CAR T cells *in vivo*. **(A)** Time line of the experimental set up. **(B)** Exemplary images of bioluminescent analysis of T cell trafficking on indicated time points. **(C)** Summary of T cell signals from each individual mouse (n=8 mouse per group; unpaired t-test*p<0.05). **(D)** Tumor growth over time for individual mice. **(E)** Kaplan-Meier-curve of mice treated with L1CAM-CAR T cells, SELPLG-deficient L1CAM-CAR T cells and untransduced T cells.

We tested the *in vivo* capacity of L1CAM-CAR T cells with and without SELPLG to infiltrate a neuroblastoma xenograft tumor generated from the SK-N-AS cell line in an immunodeficient NOG mouse model (**Figure 6A**). T cell homing to the tumor site was comparable between the two L1CAM-CAR T cell populations until day 14 after adoptive transfer (**Figure 6B**). On day 14, the median luminescent signal of SELPLG-deficient L1CAM-CAR T cells was significantly stronger in the tumor compared to L1CAM-CAR T cells retaining endogenous SELPLG expression (**Figure 6C**). Since T cell homing to the tumor site occurs within the first few days after adoptive transfer, the difference of CAR T cell signal on day 14 may not be due to better homing capacity. SELPLG-deficient L1CAM-CAR T cells were not more effective at limiting tumor growth (**Figure 6D**) or improving overall survival (**Figure 6E**) than L1CAM-CAR T cells retaining SELPLG expression. Taken together, while loss of SELPLG enhanced migratory capacity in L1CAM-CAR T cells *in vitro*, this function could not be verified in a NOG mouse model.

Since SELPLG is also described as inhibitory receptor in T cells, we analyzed whether SELPLG expression affects T cell activation and exhaustion within our experimental set up. Interestingly, enrichment analysis of gene sets related to T cell exhaustion revealed that SELPLG deficiency reduced inhibitory gene expression only in 1 out of 5 tested gene sets (**Supplementary Figure 4**). These data indicate that SELPLG might not regulate the expression of inhibitory receptors within 24 hours, or that it does not impact T cell exhaustion *in vitro*.

## 4 Discussion

Our understanding of tumor-infiltrated CAR T cells is limited by scarcity of data assessing CAR T cell trafficking through serial imaging or analysis of post-infusion biopsies. Consequently, fundamental questions to improve CAR T cell tumor infiltration and efficacy remain unanswered. Here we aimed to gain insights into the cell interactions and signaling pathways that assist migration into and through the tumor using our 3D bioprinted neuroblastoma model and L1CAM-CAR T or untransduced T cells as a simplified system. The *in vitro* setting allowed specific separation of cells that infiltrated the tumor model or remained in the culture medium, supporting both bulk and single-cell transcriptional profiling in cell groups that acted differently. Analyses from this *in vitro* set-up showed that SELPLG expression was low in T cells that entered the 3D model, while SH2D2A expression was high. Manipulating L1CAM-CAR T cells with CRISPR/Cas9 to knock out SELPLG expression or enhance SH2D2A expression did not affect CAR T cell functions necessary for anti-tumor activity *in vitro*. Only SELPLG deficiency enhanced the migratory capacity of CAR T cells *in vitro*. However, these modifications did not translate into superior tumor infiltration or enhanced anti-tumor efficacy in a NOG mouse model.

Transcriptional profiling in CAR T cells capable of entering our 3D bioprinted model indicated SH2D2A and SELPLG as candidates driving T cell migration. SH2D2A encodes the T cell-specific adaptor protein (TSAd), known to act within the CXCR4/CXCL12 signaling axis and to play a role in laminin-dependent T cell migration, assisting attachment to laminin present in tumor stroma and activating the focal adhesion kinase, a central regulator of cell migration, adhesion and survival (31, 32). Within the CXCR4/CXCL12 signaling cascade, SH2D2A primes the IL2-inducible T cell kinase (ITK) for phosphorylation by phosphinsitol-3-kinase and SRC kinase in response to CXCL12 binding by CXCR4. ITK induces actin polymerization, which is necessary for T cell migration (33–35). The role for SH2D2A in regulating CXCL12-induced T cell migration and actin cytoskeletal remodeling was demonstrated in Jurkat T cells (36). Furthermore, TSAd-deficient murine thymocytes had impaired responses to CXCL12, characterized by reduced Itk phosphorylation, diminished actin polymerization and decreased cell migration (36). We hypothesized that raising the amount of SH2D2A in the L1CAM-CAR T cells would cause more rapid migration toward the CXCL12 chemokine gradient that we synthetically re-established in the *in vitro* assay. However, enhancing SH2D2A expression did not cause more CAR T cells to migrate through an endothelial layer in a 4-hour transendothelial migration assay. These findings suggest SH2D2A does not play a key role in regulating CAR T cell tumor infiltration and migration. Targeting SH2D2A expression is unlikely to produce an improved CAR T cell product against solid tumors.

We observed elevated expression of T cell activation-related gene signatures in SH2D2A-positive cells and higher TSAd protein levels in *SH2D2A*-overexpressing T cells upon CD3/CD28 stimulation. The TSAd adapter protein function in T cell signaling also promotes LCK activation to regulate TCR-initiated cytoplasmic signaling pathways (28, 37). Within this context, our data support a mechanistic link between SH2D2A expression and T cell activation. These findings suggest that SH2D2A is closely associated with T cell activation and may serve as a T cell activation marker rather than directly promoting T cell migration.

The function of SELPLG in migration remains controversial. Earlier studies found SELPLG to be essential for T cell tethering and rolling onto selectins expressed on the endothelium (30, 38), a process preceding T cell extravasation to leave the blood vessel and enter the surrounding tissue. Most recent findings define SELPLG as a T cell inhibitory receptor and demonstrate that eliminating or blocking SELPLG promotes tumor infiltration and the capacity for T cells to migrate in immunocompetent mouse tumor models (39–42). Our findings that L1CAM-CAR T cells crossed an *in vitro* endothelial layer more rapidly after SELPLG knockout are in line with SELPLG being a negative regulator of T cell migration. Although, the mechanism behind this enhanced infiltration remains unclear. T cell numbers in melanomas harbored in Selplg-/- mice were shown to be higher and gene signatures for T cell activation and survival enhanced in previous work (39). However, we could not reproduce the improved anti-tumor efficacy and survival in our immunodeficient NOG mouse model treated with human L1CAM-CAR T cells lacking functional SELPLG. Human and murine SELPLG share 46% homology, which together with species-specific functional differences, may explain our result in the immunodeficient NOG mouse model. We can speculate that in the absence of SELPLG, an alternative pathway of T cell adhesion is exploited or that SELPLG is molecularly redundant in T cell migration and some other protein takes over its role. In mice for example, Selectins P and E (Selp and Sele, respectively) have overlapping and partially redundant functions, indicating that a further ligand for these selectins exists, at least in mice (43, 44). Selectin P (SELP) is the only receptor for SELPLG (45) known to date in humans. It is possible that SELPLG expression may increase T cell retention. While SELPLG is important for initial leukocyte tethering and rolling (30, 38, 45), it can also promote adhesion and retention (46). High SELPLG expression might act as a “brake” to T cell movement. This slowing or even arresting of T cell migration would prolong their interactions with endothelial or stromal selectins. SELPLG-deficient T cells might, therefore, transit more freely into the tumor because they are not retained at vessel walls or stromal structures. SELPLG has been described as an inhibitory receptor (41, 42, 47). While it is possible that SELPLG could promote a terminally exhausted T cell state that is less migratory and less responsive to chemokines, exhaustion-related genes were not upregulated in SELPLG-positive cells in our data set, which does not support this option.

We employed the NOG mouse to assess SELPLG-deficient L1CAM-CAR T cell migration *in vivo* because it’s a common model used in CAR T cell studies. It enables engraftment of human tumor and (CAR) T cells. A deficiency of the NOG model is that it does not provide the human cytokine support necessary for optimal T cell function. The low homology between the human and murine SELPLG protein presents a further deficiency that may limit the translatability of the *in vivo* findings between mice and humans. This limitation may have underestimated L1CAM-CAR T cell migratory capacity in a human tumor, as well as their true therapeutic potential and efficacy in humans. Our findings here indicate that mouse models are unsuited for discovery purposes of specific ligand–receptor interactions involved in mechanisms for T cell tumor invasion. Mouse models may not be able to replicate certain mechanistic features of human T cell migration into tumors. Here, we cannot exclude a potential role for SELPLG in human CAR T cell migration and invasion under more physiologically relevant conditions.

We conclude from our findings that our basic bioprinted 3D tumor model is appropriate for the purposes of initial screening. It provides a rapid, cost-effective and reproducible system that can serve as a functional filter to identify genes relevant to T cell migration. We identified a set of candidate genes potentially involved in this process using RNA sequencing. From these, five genes were prioritized based on expression profiles and putative functional roles, and the top two, SELPLG and SH2D2A, were selected for experimental validation. SELPLG emerged as the most promising candidate *in vitro* but failed our subsequent *in vivo* evaluation. For more detailed mechanistic studies or to evaluate the impact of complex tumor microenvironment interactions, the use of a more advanced and physiologically representative model will be essential. A more advanced fully human 3D tumor model would better support validation of the migration-promoting function of SELPLG. To better reflect tumor complexity, such models should incorporate immunosuppressive factors, stromal elements and the structural barriers characteristic of solid tumors in patients. Recent developments, such as the synthetic generation of vascular structures within bioprinted tumor models (48) and the incorporation of spatially organized tumor microenvironment components, including fibroblasts and regulatory immune populations (49), highlight the potential of more physiologically relevant bioprintable 3D tumor and tissue models. Despite these advances, current *in vitro* and murine model systems still fail to fully represent the cellular diversity and the spatial organization of human tumors, underscoring the urgent need for next-generation 3D fully human tumor models. Development of these models is essential to accurately predict therapeutic responses and guide the design of immune cell-based therapies, such as CAR T cells. Discovery and validation of receptor–ligand interactions active in the mechanisms controlling T cell tumor infiltration and migration within the tumor would be highly supported by these new entirely human 3D tumor and tissue models. We expect this innovation to assist research to improve CAR T cell efficacy.

Here, we demonstrate that SELPLG-deficient L1CAM-CAR T cells exhibit enhanced migratory capacity *in vitro* without compromising CAR T cell functionality. However, these *in vitro* results did not translate to increased anti-tumor efficacy in an immunodeficient mouse model. This discrepancy underscores the complexity of translating *in vitro* findings into improvement for CAR T cell therapies. Our results support advancing all-human complex 3D tumor models to incorporate the physiologically relevant structures and cell populations as better models than mice to advance CAR T cell development and validation.

## Data Availability

The datasets presented in this study can be found in online repositories. The names of the repository/repositories and accession number(s) can be found below: https://figshare.com/, DOI 10.6084/m9.figshare.29624960.

## Supplementary material

Supplementary Figure 1 **(A)** Principal component analysis (PCA) of RNA libraries of each sample. Each dot represents gene expression of infiltrated and non-infiltrated L1CAM-CAR T cells harboring the 4-1BB (brown and yellow) or the CD28 (pink and grey) co-stimulatory domain or untransduced T cells (turquoise and green). **(B, C)** Gene set enrichment analysis of MSigDB C2 data sets **(B)** and gene sets derived from the gene ontology term “biological process” **(C)** upregulated in 3D-tumor infiltrated (CAR) T cells following 24 h coculture. **(D)** Volcano plot of differentially expressed genes in L1CAM-CAR T cells (CD28 and 4-1BB costimulated) isolated from 3D tumor model versus non-infiltrated CAR T cells. J. Volcano plot showing DEG between 3D tumor model-infiltrated L1CAM-CAR T cells harboring the CD28 versus 4-1BB co-stimulatory domain.

Supplementary Figure 2 Distribution of cells expressing the gene sets of GO term “lymphocyte migration” **(A)** and “regulation of lymphocyte migration” retrieved from MSigDB gene set C5 **(B)** within the UMAP embedding. Violin Plots show comparing gene expression between different T cell clusters. **(C, D)** Tumor-infiltrated untransduced and CAR T cells were categorized in SH2D2A- **(C)** and SELPLG- **(D)** positive and negative cells. Violin plots show expression of indicated genes in all T cell subsets. E. Gene set enrichment analysis of indicated T cell subsets. Gene sets on T cell activation and T cell exhaustion were analyzed. Kruskal-Wallis test, ns: p>0.05, *p<=0.05, **p<=0.01, ***p<=0.001, ****p<= 0.0001.

Supplementary Figure 3 **(A)** Full image of Westernblot gel. SH2D2A overexpression in untreated T cells (control) and after 24 h anti-CD3/CD28 stimulation using anti-SH2D2A and anti-GFP antibody. GAPDH served as loading control. Westernblot images were captured in two sequential steps at the same membrane position and orientation. Molecular weight marker (ladder) was imaged prior to substrate application and subsequent chemiluminescent detection of protein bands. The marker image was then overlaid onto the chemiluminescent image using VisionCapt_v16.16d software (Fusion system). **(B, C)** Exemplary dot plot of flow cytometry analysis showing GFP expression **(B)** or cell surface expression of CXCR4 **(C)** of the different T cell subtypes cells before experiment (flask) and after 2 h and 4 h upon start of transwell migration assay in the migrated cell fraction. Gating was applied for living cells.

Supplementary Figure 4 **(A)** SELPLG expression in untransduced (UT), L1CAM-CAR-T cells (CAR) and SELPLG-deficient L1CAM-CAR T cells (CAR^SELPLG-KO^) as measured via flow cytometry. **(B)** Tumor-infiltrated untransduced and CAR T cells were categorized in SELPLG- positive and negative cells. Gene set enrichment analysis of indicated T cell were analyzed. Kruskal-Wallis test, ns: p>0.05, *p<=0.05, **p<=0.01, ***p<=0.001, ****p<= 0.0001.

## References

[1] Maris JM . Recent Advances in Neuroblastoma. New Engl J Med. (2010) 362:2202–11. doi: 10.1056/NEJMra0804577, PMID: 20558371 PMC3306838

[2] Smith MA Altekruse SF Adamson PC Reaman GH Seibel NL . Declining Childhood and Adolescent Cancer Mortality. Cancer. (2014) 120:2497–506. doi: 10.1002/cncr.28748, PMID: 24853691 PMC4136455

[3] Louis CU Savoldo B Dotti G Pule M Yvon E Myers GD . Antitumor Activity and Long-Term Fate of Chimeric Antigen Receptor-Positive T Cells in Patients with Neuroblastoma. Blood. (2011) 118:6050–6. doi: 10.1182/blood-2011-05-354449, PMID: 21984804 PMC3234664

[4] Del Bufalo F De Angelis B Caruana I Del Baldo G De Ioris MA Serra A . Gd2-Cart01 for Relapsed or Refractory High-Risk Neuroblastoma. New Engl J Med. (2023) 388:1284–95. doi: 10.1056/NEJMoa2210859, PMID: 37018492

[5] Straathof K Flutter B Wallace R Jain N Loka T Depani S . Antitumor Activity without on-Target Off-Tumor Toxicity of Gd2–Chimeric Antigen Receptor T Cells in Patients with Neuroblastoma. Sci Transl Med. (2020), 12:1–12. doi: 10.1126/scitranslmed.abd6169, PMID: 33239386

[6] Heczey A Louis CU Savoldo B Dakhova O Durett A Grilley B . Car T Cells Administered in Combination with Lymphodepletion and Pd-1 Inhibition to Patients with Neuroblastoma. Mol Ther. (2017) 25:2214–24. doi: 10.1016/j.ymthe.2017.05.012, PMID: 28602436 PMC5589058

[7] Alon R Kassner PD Carr MW Finger EB Hemler ME Springer TA . The Integrin Vla-4 Supports Tethering and Rolling in Flow on Vcam-1. J Cell Biol. (1995) 128:1243–53. doi: 10.1083/jcb.128.6.1243, PMID: 7534768 PMC2120426

[8] Berlin C Bargatze RF Campbell JJ von Andrian UH Szabo MC Hasslen SR . α4 Integrins Mediate Lymphocyte Attachment and Rolling under Physiologic Flow. Cell. (1995) 80:413–22. doi: 10.1016/0092-8674(95)90491-3, PMID: 7532110

[9] DeGrendele HC Estess P Picker LJ Siegelman MH . Cd44 and Its Ligand Hyaluronate Mediate Rolling under Physiologic Flow: A Novel Lymphocyte-Endothelial Cell Primary Adhesion Pathway. J Exp Med. (1996) 183:1119–30. doi: 10.1084/jem.183.3.1119, PMID: 8642254 PMC2192320

[10] Soriano SF Hons M Schumann K Kumar V Dennier TJ Lyck R . *In Vivo* Analysis of Uropod Function During Physiological T Cell Trafficking. J Immunol. (2011) 187:2356–64. doi: 10.4049/jimmunol.1100935, PMID: 21795598

[11] Jacobelli J Estin Matthews M Chen S Krummel MF . Activated T Cell Trans-Endothelial Migration Relies on Myosin-Iia Contractility for Squeezing the Cell Nucleus through Endothelial Cell Barriers. PloS One. (2013) 8:e75151. doi: 10.1371/journal.pone.0075151, PMID: 24069389 PMC3777879

[12] Krummel MF Bartumeus F Gerard A . T Cell Migration, Search Strategies and Mechanisms. Nat Rev Immunol. (2016) 16:193–201. doi: 10.1038/nri.2015.16, PMID: 26852928 PMC4869523

[13] Kershaw MH Westwood JA Parker LL Wang G Eshhar Z Mavroukakis SA . A Phase I Study on Adoptive Immunotherapy Using Gene-Modified T Cells for Ovarian Cancer. Clin Cancer Res. (2006) 12:6106–15. doi: 10.1158/1078-0432.CCR-06-1183, PMID: 17062687 PMC2154351

[14] Papa S Adami A Metoudi M Beatson R George MS Achkova D . Intratumoral Pan-Erbb Targeted Car-T for Head and Neck Squamous Cell Carcinoma: Interim Analysis of the T4 Immunotherapy Study. J Immunother Cancer. (2023) 11:1–13. doi: 10.1136/jitc-2023-007162, PMID: 37321663 PMC10277526

[15] Majzner RG Ramakrishna S Yeom KW Patel S Chinnasamy H Schultz LM . Gd2-Car T Cell Therapy for H3k27m-Mutated Diffuse Midline Gliomas. Nature. (2022) 603:934–41. doi: 10.1038/s41586-022-04489-4, PMID: 35130560 PMC8967714

[16] Narayan V Barber-Rotenberg JS Jung IY Lacey SF Rech AJ Davis MM . Psma-Targeting Tgfbeta-Insensitive Armored Car T Cells in Metastatic Castration-Resistant Prostate Cancer: A Phase 1 Trial. Nat Med. (2022) 28:724–34. doi: 10.1038/s41591-022-01726-1, PMID: 35314843 PMC10308799

[17] Künkele A Taraseviciute A Finn LS Johnson AJ Berger C Finney O . Preclinical Assessment of Cd171-Directed Car T-Cell Adoptive Therapy for Childhood Neuroblastoma: Ce7 Epitope Target Safety and Product Manufacturing Feasibility. Clin Cancer Res. (2017) 23:466–77. doi: 10.1158/1078-0432.CCR-16-0354, PMID: 27390347

[18] Grunewald L Lam T Andersch L Klaus A Schwiebert S Winkler A . A Reproducible Bioprinted 3d Tumor Model Serves as a Preselection Tool for Car T Cell Therapy Optimization. Front Immunol. (2021) 12:689697. doi: 10.3389/fimmu.2021.689697, PMID: 34267756 PMC8276678

[19] Kunkele A Johnson AJ Rolczynski LS Chang CA Hoglund V Kelly-Spratt KS . Functional Tuning of Cars Reveals Signaling Threshold above Which Cd8+ Ctl Antitumor Potency Is Attenuated Due to Cell Fas-Fasl-Dependent Aicd. Cancer Immunol Res. (2015) 3:368–79. doi: 10.1158/2326-6066.CIR-14-0200, PMID: 25576337

[20] Ausubel LJ Hall C Sharma A Shakeley R Lopez P Quezada V . Production of Cgmp-Grade Lentiviral Vectors. Bioprocess Int. (2012) 10:32–43., PMID: 22707919 PMC3374843

[21] Wang X Chang WC Wong CLW Colcher D Sherman M Ostberg JR . A Transgene-Encoded Cell Surface Polypeptide for Selection, *in Vivo* Tracking, and Ablation of Engineered Cells. Blood. (2011) 118:1255–63. doi: 10.1182/blood-2011-02-337360, PMID: 21653320 PMC3152493

[22] Wang X Narajano A Brown C Bautista C Wong C Chang W-C . Phenotypic and Functional Attributes of Lentivirus Modified Cd19-Specific Human Cd8+ Central Memory T Cells Manufactured at Clinical Scale. J Immunother. (2012) 35:689–701. doi: 10.1097/CJI.0b013e318270dec7.Phenotypic, PMID: 23090078 PMC3525345

[23] Zheng L Qin S Si W Wang A Xing B Gao R . Pan-Cancer Single-Cell Landscape of Tumor-Infiltrating T Cells. Science. (2021) 374:abe6474. doi: 10.1126/science.abe6474, PMID: 34914499

[24] Charo IF Ransohoff RM . The Many Roles of Chemokines and Chemokine Receptors in Inflammation. New Engl J Med. (2006) 354:610–21. doi: 10.1056/NEJMra052723, PMID: 16467548

[25] Worbs T Mempel TR Bölter J von Andrian UH Förster R . Ccr7 Ligands Stimulate the Intranodal Motility of T Lymphocytes *in Vivo*. J Exp Med. (2007) 204:489–95. doi: 10.1084/jem.20061706, PMID: 17325198 PMC2137901

[26] Walling BL Kim M . Lfa-1 in T Cell Migration and Differentiation. Front Immunol. (2018) 9:952. doi: 10.3389/fimmu.2018.00952, PMID: 29774029 PMC5943560

[27] Granum S Sundvold-Gjerstad V Dai KZ Kolltveit KM Hildebrand K Huitfeldt HS . Structure Function Analysis of Sh2d2a Isoforms Expressed in T Cells Reveals a Crucial Role for the Proline Rich Region Encoded by Sh2d2a Exon 7. BMC Immunol. (2006) 7:15. doi: 10.1186/1471-2172-7-15, PMID: 16839418 PMC1553471

[28] Marti F Garcia GG Lapinski PE MacGregor JN King PD . Essential Role of the T Cell-Specific Adapter Protein in the Activation of Lck in Peripheral T Cells. J Exp Med. (2006) 203:281–7. doi: 10.1084/jem.20051637, PMID: 16446380 PMC2118198

[29] Marti F Post NH Chan E King PD . A Transcription Function for the T Cell–Specific Adapter (Tsad) Protein in T Cells: Critical Role of the Tsad Src Homology 2 Domain. J Exp Med. (2001) 193:1425–30. doi: 10.1084/jem.193.12.1425, PMID: 11413197 PMC2193301

[30] Asaduzzaman M Mihaescu A Wang Y Sato T Thorlacius H . P-Selectin and P-Selectin Glycoprotein Ligand 1 Mediate Rolling of Activated Cd8+ T Cells in Inflamed Colonic Venules. J Invest Med. (2009) 57:765–8. doi: 10.231/JIM.0b013e3181b918fb, PMID: 19730132

[31] Park E Choi Y Ahn E Park I Yun Y . The Adaptor Protein Lad Tsad Mediates Laminin-Dependent T Cell Migration Via Association with the 67 Kda Laminin Binding Protein. Exp Mol Med. (2009) 41:728–36. doi: 10.3858/emm.2009.41.10.079, PMID: 19561400 PMC2772975

[32] Parsons JT Martin KH Slack JK Taylor JM Weed SA . Focal Adhesion Kinase: A Regulator of Focal Adhesion Dynamics and Cell Movement. Oncogene. (2000) 19:5606–13. doi: 10.1038/sj.onc.1203877, PMID: 11114741

[33] Fischer AM Mercer JC Iyer A Ragin MJ August A . Regulation of Cxc Chemokine Receptor 4-Mediated Migration by the Tec Family Tyrosine Kinase Itk. J Biol Chem. (2004) 279:29816–20. doi: 10.1074/jbc.M312848200, PMID: 15123627

[34] Sundvold V Torgersen KM Post NH Marti F King PD Rottingen JA . T Cell-Specific Adapter Protein Inhibits T Cell Activation by Modulating Lck Activity. J Immunol. (2000) 165:2927–31. doi: 10.4049/jimmunol.165.6.2927, PMID: 10975797

[35] Park D Park I Lee D Choi YB Lee H Yun Y . The Adaptor Protein Lad Associates with the G Protein Beta Subunit and Mediates Chemokine-Dependent T-Cell Migration. Blood. (2007) 109:5122–8. doi: 10.1182/blood-2005-10-061838, PMID: 17327418

[36] Berge T Sundvold-Gjerstad V Granum S Andersen TCB Holthe GB Claesson-Welsh L . T Cell Specific Adapter Protein (Tsad) Interacts with Tec Kinase Itk to Promote Cxcl12 Induced Migration of Human and Murine T Cells. PloS One. (2010) 5:1–10. doi: 10.1371/journal.pone.0009761, PMID: 20305788 PMC2841202

[37] Kolltveit KM Granum S Aasheim HC Forsbring M Sundvold-Gjerstad V Dai KZ . Expression of Sh2d2a in T-Cells Is Regulated Both at the Transcriptional and Translational Level. Mol Immunol. (2008) 45:2380–90. doi: 10.1016/j.molimm.2007.11.005, PMID: 18160104

[38] Snapp KR Heitzig CE Kansas GS . Attachment of the Psgl-1 Cytoplasmic Domain to the Actin Cytoskeleton Is Essential for Leukocyte Rolling on P-Selectin. Blood. (2002) 99:4494–502. doi: 10.1182/blood.V99.12.4494, PMID: 12036880

[39] DeRogatis JM Viramontes KM Neubert EN Henriquez ML Guerrero-Juarez CF Tinoco R . Targeting the Psgl-1 Immune Checkpoint Promotes Immunity to Pd-1-Resistant Melanoma. Cancer Immunol Res. (2022) 10:612–25. doi: 10.1158/2326-6066.CIR-21-0690, PMID: 35303066 PMC9064985

[40] Viramontes KM Neubert EN DeRogatis JM Tinoco R . Pd-1 Immune Checkpoint Blockade and Psgl-1 Inhibition Synergize to Reinvigorate Exhausted T Cells. Front Immunol. (2022) 13:869768. doi: 10.3389/fimmu.2022.869768, PMID: 35774790 PMC9237324

[41] Matsumoto M Miyasaka M Hirata T . P-Selectin Glycoprotein Ligand-1 Negatively Regulates T-Cell Immune Responses. J Immunol. (2009) 183:7204–11. doi: 10.4049/jimmunol.0902173, PMID: 19890058

[42] Tinoco R Otero DC Takahashi A Brandley LM . Psgl-1: A New Player in the Immune Checkpoint Landscape. Trends Immunol. (2017) 38:323–35. doi: 10.1016/j.it.2017.02.002, PMID: 28262471 PMC5411281

[43] Tietz W Allemand Y Borges E von Laer D Hallmann R Vestweber D . Cd4+ T Cells Migrate into Inflamed Skin Only If They Express Ligands for E- and P-Selectin. J Immunol. (1998) 161:963–70. doi: 10.4049/jimmunol.161.2.963, PMID: 9670976

[44] Yamada S Mayadas TN Yuan F Wagner DD Hynes RO Melder RJ . Rolling in P-Selectin-Deficient Mice Is Reduced but Not Eliminated in the Dorsal Skin. Blood. (1995) 86:3487–92. doi: 10.1182/blood.V86.9.3487.bloodjournal8693487, PMID: 7579454

[45] Matsumoto M Shigeta A Furukawa Y Tanaka T Miyasaka M Hirata T . Cd43 Collaborates with P-Selectin Glycoprotein Ligand-1 to Mediate E-Selectin-Dependent T Cell Migration into Inflamed Skin. J Immunol. (2007) 178:2499–506. doi: 10.4049/jimmunol.178.4.2499, PMID: 17277158

[46] Battistini L Piccio L Rossi B Bach S Galgani S Gasperini C . Cd8+ T Cells from Patients with Acute Multiple Sclerosis Display Selective Increase of Adhesiveness in Brain Venules:A Critical Role for P-Selectin Glycoprotein Ligand-1. Blood. (2003) 101:4775–82. doi: 10.1182/blood-2002-10-3309, PMID: 12595306

[47] Tinoco R Carrette F Barraza ML Otero DC Magana J Bosenberg MW . Psgl-1 Is an Immune Checkpoint Regulator That Promotes T Cell Exhaustion. Immunity. (2016) 44:1190–203. doi: 10.1016/j.immuni.2016.04.015, PMID: 27192578 PMC4908967

[48] Sexton ZA-O Rütsche DA-OX Herrmann JA-O Hudson AA-O Sinha S Du JA-O . Rapid Model-Guided Design of Organ-Scale Synthetic Vasculature for Biomanufacturing. Science. (2025) 388:1198–204. doi: 10.1126/science.adj6152, PMID: 40504910 PMC12490288

[49] Jiang Y Jin L Liu W Liu H Liu X Tan Z . Construction of 3d Tumor *in Vitro* Models with an Immune Microenvironment Exhibiting Similar Tumor Properties and Biomimetic Physiological Functionality. Biomater Sci. (2024) 13:223–35. doi: 10.1039/d4bm00754a, PMID: 39526532

[50] Vicente-Manzanares M Ma X Adelstein RS Horwitz AR . Non-Muscle Myosin Ii Takes Centre Stage in Cell Adhesion and Migration. Nat Rev Mol Cell Biol. (2009) 10:778–90. doi: 10.1038/nrm2786, PMID: 19851336 PMC2834236

[51] Juanes-Garcia A Chapman JR Aguilar-Cuenca R Delgado-Arevalo C Hodges J Whitmore LA . A Regulatory Motif in Nonmuscle Myosin Ii-B Regulates Its Role in Migratory Front-Back Polarity. J Cell Biol. (2015) 209:23–32. doi: 10.1083/jcb.201407059, PMID: 25869664 PMC4395487

[52] Chen JS Su IJ Leu YW Young KC Sun HS . Expression of T-Cell Lymphoma Invasion and Metastasis 2 (Tiam2) Promotes Proliferation and Invasion of Liver Cancer. Int J Cancer. (2012) 130:1302–13. doi: 10.1002/ijc.26117, PMID: 21469146

[53] Woroniuk A Porter A White G Newman DT Diamantopoulou Z Waring T . Stef/Tiam2-Mediated Rac1 Activity at the Nuclear Envelope Regulates the Perinuclear Actin Cap. Nat Commun. (2018) 9:2124. doi: 10.1038/s41467-018-04404-4, PMID: 29844364 PMC5974301

[54] Zhao ZY Han CG Liu JT Wang CL Wang Y Cheng LY . Tiam2 Enhances Non-Small Cell Lung Cancer Cell Invasion and Motility. Asian Pac J Cancer Prev. (2013) 14:6305–9. doi: 10.7314/apjcp.2013.14.11.6305, PMID: 24377522

[55] Cai L Marshall T Uetrecht A Schafer D Bear J . Coronin 1b Coordinates Arp2/3 Complex and Cofilin Activities at the Leading Edge. Cell. (2007) 128:915–29. doi: 10.1016/j.cell.2007.01.031, PMID: 17350576 PMC2630706

[56] Ojeda V Castro-Castro A Bustelo XR . Coronin1 Proteins Dictate Rac1 Intracellular Dynamics and Cytoskeletal Output. Mol Cell Biol. (2014) 34:3388–406. doi: 10.1128/MCB.00347-14, PMID: 24980436 PMC4135624

[57] Rana M Worthylake R . Novel Mechanism for Negatively Regulating Rho-Kinase (Rock) Signaling through Coronin1b Protein in Neuregulin 1 (Nrg-1)-Induced Tumor Cell Motility. J Biol Chem. (2012) 287:21836–45. doi: 10.1074/jbc.M112.346114, PMID: 22563075 PMC3381146

[58] Yan M Di Ciano-Oliveira C Grinstein S Trimble WS . Coronin Function Is Required for Chemotaxis and Phagocytosis in Human Neutrophils. J Immunol. (2007) 178:5769–78. doi: 10.4049/jimmunol.178.9.5769, PMID: 17442961

[59] Bahbouhi B Berthelot L Pettré S Michel L Wiertlewski S Weksler B . Peripheral Blood Cd4 T Lymphocytes from Multiple Sclerosis Patients Are Characterized by Higher Psgl-1 Expression and Transmigration Capacity across a Human Blood-Brain Barrier-Derived Endothelial Cell Line. J Leucocyte Biol. (2009) 86:1049–63. doi: 10.1189/jlb.1008666, PMID: 19696154

[60] Xiao B Tong C Jia X Guo R Lü S Zhang Y . Tyrosine Replacement of Psgl-1 Reduces Association Kinetics with P- and L-Selectin on the Cell Membrane. Biophys J. (2012) 103:777–85. doi: 10.1016/j.bpj.2012.07.028, PMID: 22947939 PMC3443791

