# Supplementary figures and images for: Investigating genetic modifications to enhance L1CAM-CAR T cell migration in solid tumors in a 3D bioprinted neuroblastoma model
Source: Front Immunol. 2025 Nov 27;16:1677361. doi: 10.3389/fimmu.2025.1677361 (PMC12695837; doi:10.3389/fimmu.2025.1677361)

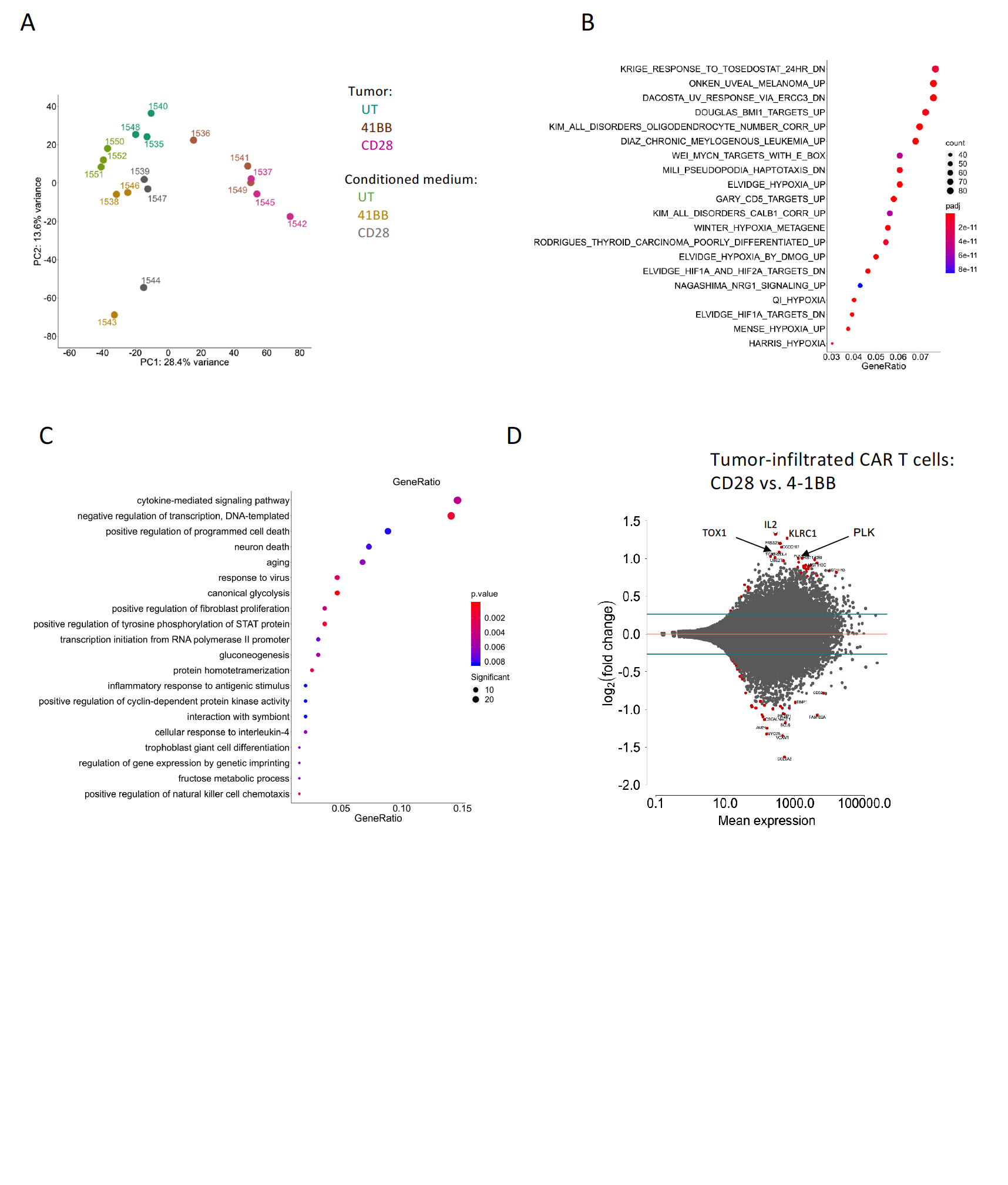

Supplement: Supplementary Figure 1 — (A) Principal component analysis (PCA) of RNA libraries of each sample. Each dot represents gene expression of infiltrated and non-infiltrated L1CAM-CAR T cells harboring the 4-1BB (brown and yellow) or the CD28 (pink and grey) co-stimulatory domain or untransduced T cells (turquoise and green). (B, C) Gene set enrichment analysis of MSigDB C2 data sets (B) and gene sets derived from the gene ontology term “biological process” (C) upregulated in 3D-tumor infiltrated (CAR) T cells following 24 h coculture. (D) Volcano plot of differentially expressed genes in L1CAM-CAR T cells (CD28 and 4-1BB costimulated) isolated from 3D tumor model versus non-infiltrated CAR T cells. J. Volcano plot showing DEG between 3D tumor model-infiltrated L1CAM-CAR T cells harboring the CD28 versus 4-1BB co-stimulatory domain. [file Image1.tiff]

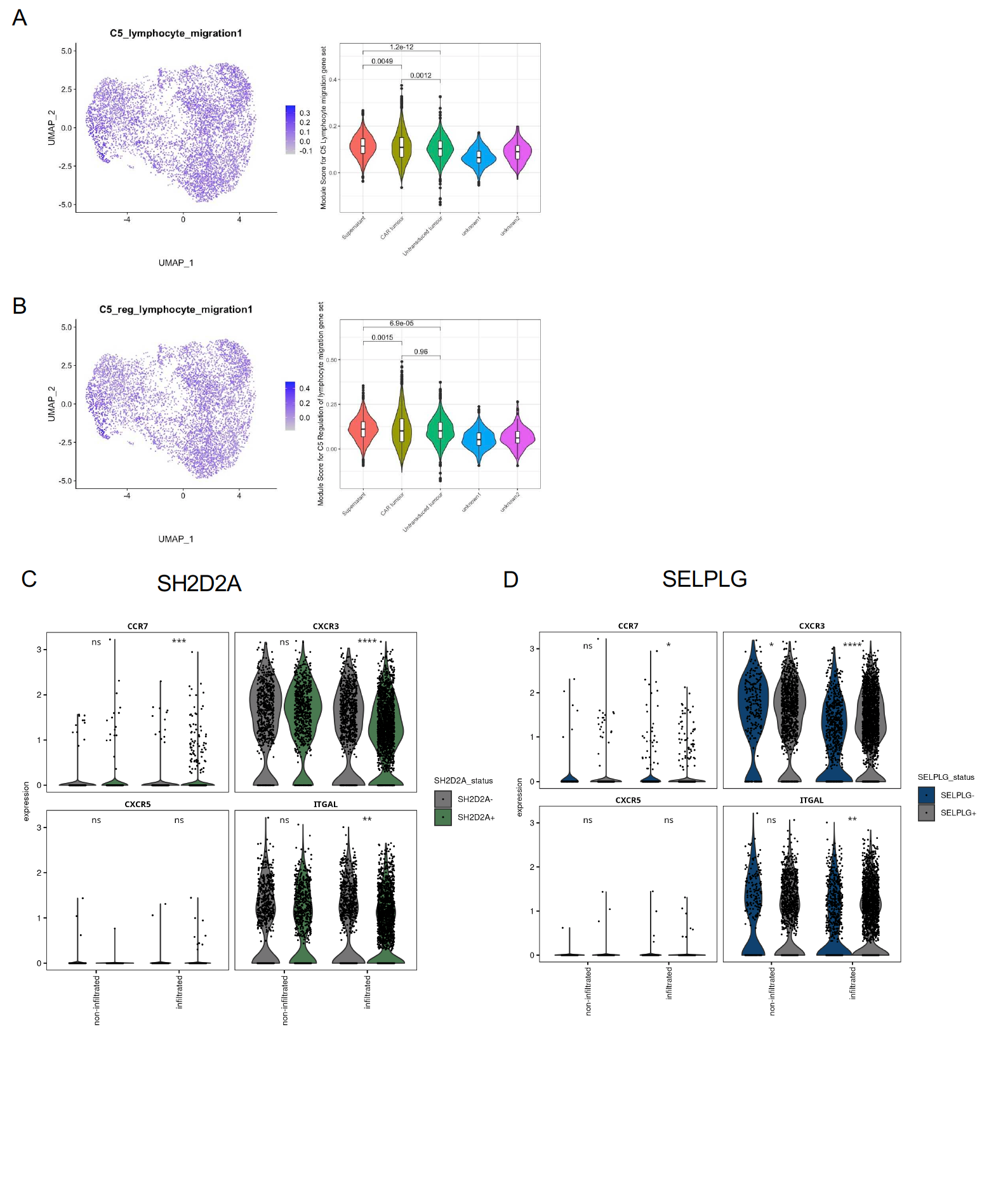

Supplement: Supplementary Figure 2 — Distribution of cells expressing the gene sets of GO term “lymphocyte migration” (A) and “regulation of lymphocyte migration” retrieved from MSigDB gene set C5 (B) within the UMAP embedding. Violin Plots show comparing gene expression between different T cell clusters. (C, D) Tumor-infiltrated untransduced and CAR T cells were categorized in SH2D2A- (C) and SELPLG- (D) positive and negative cells. Violin plots show expression of indicated genes in all T cell subsets. E. Gene set enrichment analysis of indicated T cell subsets. Gene sets on T cell activation and T cell exhaustion were analyzed. Kruskal-Wallis test, ns: p>0.05, *p<=0.05, **p<=0.01, ***p<=0.001, ****p<= 0.0001. [file Image2.png]

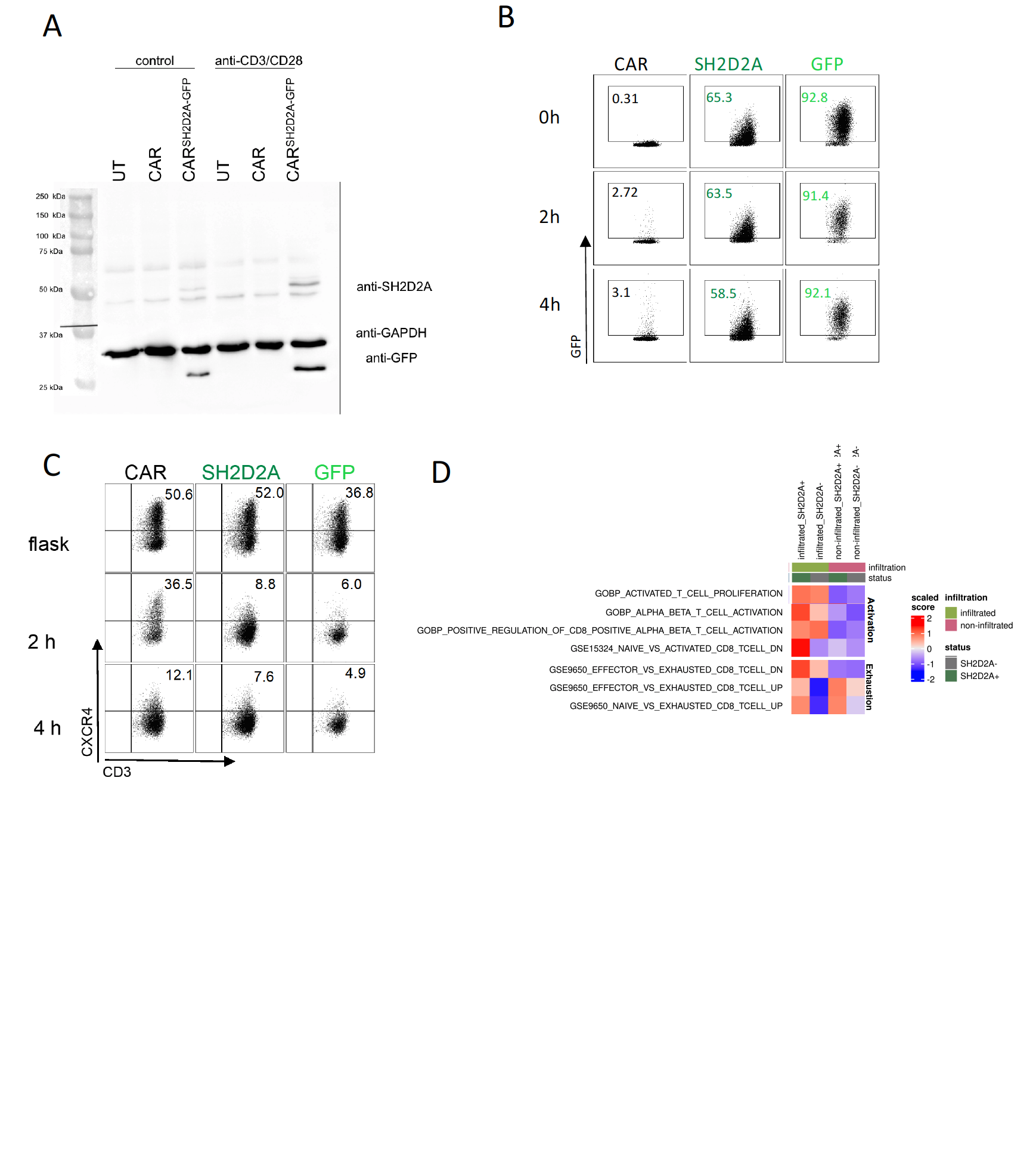

Supplement: Supplementary Figure 3 — (A) Full image of Westernblot gel. SH2D2A overexpression in untreated T cells (control) and after 24 h anti-CD3/CD28 stimulation using anti-SH2D2A and anti-GFP antibody. GAPDH served as loading control. Westernblot images were captured in two sequential steps at the same membrane position and orientation. Molecular weight marker (ladder) was imaged prior to substrate application and subsequent chemiluminescent detection of protein bands. The marker image was then overlaid onto the chemiluminescent image using VisionCapt_v16.16d software (Fusion system). (B, C) Exemplary dot plot of flow cytometry analysis showing GFP expression (B) or cell surface expression of CXCR4 (C) of the different T cell subtypes cells before experiment (flask) and after 2 h and 4 h upon start of transwell migration assay in the migrated cell fraction. Gating was applied for living cells. [file Image3.tiff]

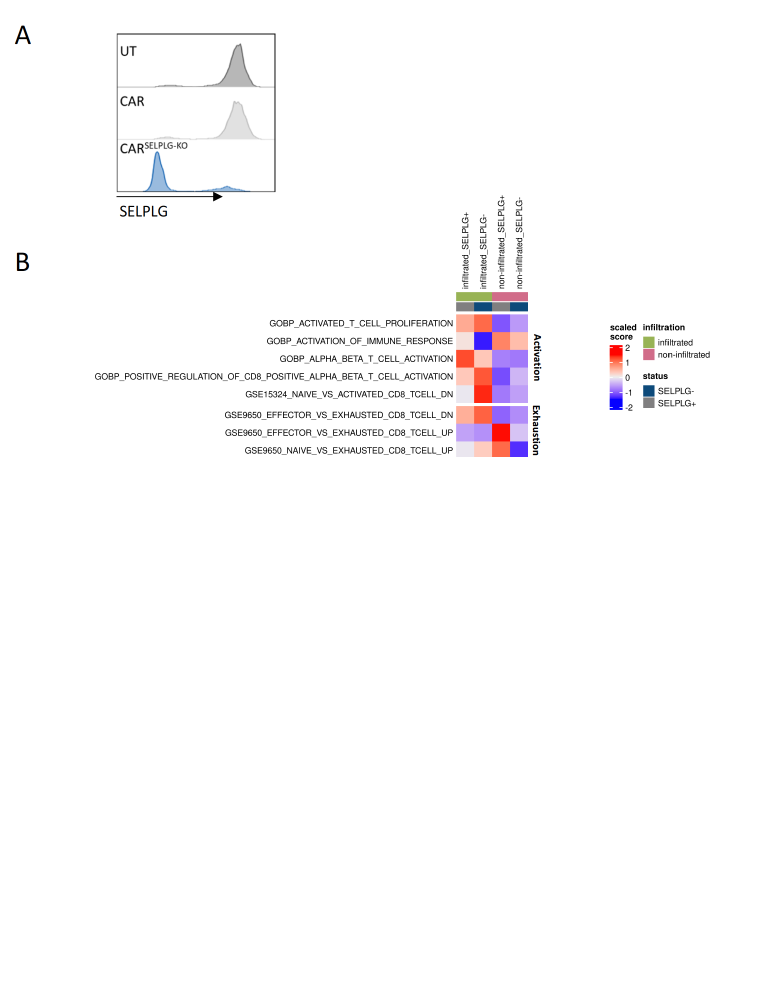

Supplement: Supplementary Figure 4 — (A) SELPLG expression in untransduced (UT), L1CAM-CAR-T cells (CAR) and SELPLG-deficient L1CAM-CAR T cells (CARSELPLG-KO) as measured via flow cytometry. (B) Tumor-infiltrated untransduced and CAR T cells were categorized in SELPLG- positive and negative cells. Gene set enrichment analysis of indicated T cell were analyzed. Kruskal-Wallis test, ns: p>0.05, *p<=0.05, **p<=0.01, ***p<=0.001, ****p<= 0.0001. [file Image4.tiff]
